# Tumor Microenvironment‐Activated Nanocomposite for Self‐Amplifying Chemodynamic/Starvation Therapy Enhanced IDO‐Blockade Tumor Immunotherapy

**DOI:** 10.1002/advs.202303580

**Published:** 2023-10-09

**Authors:** Yulong Bian, Bin Liu, Binbin Ding, Meifang Wang, Meng Yuan, Ping'an Ma, Jun Lin

**Affiliations:** ^1^ State Key Laboratory of Rare Earth Resource Utilization Changchun Institute of Applied Chemistry Chinese Academy of Sciences Changchun 130022 China; ^2^ School of Applied Chemistry and Engineering University of Science and Technology of China Hefei 230026 China

**Keywords:** chemodynamic/starvation therapy, immunogenic cell death, immunotherapy, indoleamine 2,3‐dioxygenase, tumor microenvironment‐responsive

## Abstract

Disrupting intracellular redox homeostasis combined with immune checkpoint blockade therapy is considered as an effective way to accelerate tumor cell death. However, suppressed tumor immune microenvironment and lower cargo delivery restrict the efficiency of tumor therapy. In this study, a multifunctional tumor microenvironment (TME)‐responsive nanocomposite is constructed using manganese tetroxide (Mn_3_O_4_)‐decorated disulfide‐bond‐incorporated dendritic mesoporous organosilica nanoparticles (DMONs) to co‐deliver indoleamine 2,3‐dioxygenase (IDO) inhibitor Epacadostat (IDOi) and glucose oxidase (GOx) following modification with polyethylene glycol. Owing to the responsiveness of Mn_3_O_4_‐decorated DMONs to the mildly acidic and glutathione (GSH) overexpressed TME, the nanocomposite can rapidly decompose and release inner contents, thus substantially improving the cargo release ability. Mn_3_O_4_ can effectively catalyze hydrogen peroxide (H_2_O_2_) decomposition to generate oxygen, enhance the ability of GOx to consume glucose to produce H_2_O_2_, and further promote the generation of hydroxyl radicals (•OH) by Mn^2+^. Furthermore, Mn^2+^‐mediated GSH depletion and the production of •OH can disrupt intracellular redox homeostasis, contributing to immunogenic cell death. Simultaneously, IDOi can inhibit IDO to reverse inhibited immune response. The results show that self‐amplifying chemodynamic/starvation therapy combined IDO‐blockade immunotherapy synergistically inhibits tumor growth and metastasis in vivo.

## Graphical abstract

Here, the large‐pore organosilica mesoporous‐silica decorated Mn_3_O_4_ nanoparticles as the cornerstone are prepared first. Then by loading IDOi, GOx, and modifying the DSPE‐PEG. The self‐amplification chemodynamic/starvation therapy can enhance immunogenic cell death, while IDOi inhibit the IDO activity. Therefore, the successfully constructed MnDIG@PEG nanocomposite can inhibit tumor growth and metastasis through TME‐responsive self‐amplifying multimodal therapeutic strategies for oncotherapy.

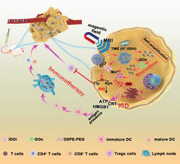

## 1 Introduction

With the emergence of nanotherapeutics, the ever‐increasing focus consists of the external noninvasive and accurate oncotherapy.^[^ ^1^ ^]^ Chemodynamic therapy (CDT), which involves the use of various transition metal ions to catalyze the endogenous hydrogen peroxide (H_2_O_2_) through Fenton or Fenton‐like reactions to generate highly cytotoxic hydroxyl radicals (•OH) to eliminate tumor cells, is attracting considerable attention owing to its high therapeutic performance in deep‐tissue hypoxic tumor therapy with little damage to normal tissue.^[^ ^2^ ^]^ The generated •OH during CDT can increase the exposure of tumor‐associated antigens to induce immunogenic cell death (ICD).^[^ ^3^ ^]^ However, the cytotoxic •OH can be partially eliminated by the overexpressed glutathione (GSH) in the tumor microenvironment (TME) before the onset of its action.^[^ ^4^ ^]^ Additionally, Fe^2+^‐catalyzed CDT, the most common CDT technique, is usually activated in highly acidic environments (pH < 4).^[^ ^5^ ^]^ In contrast, Mn^2+^‐catalyzed CDT can kill tumor cells even in only slightly acidic environments (pH 5), therefore holding great superiority as a CDT catalyzer for oncotherapy.^[^ ^6^ ^]^ Simultaneously, the conversion of redox pairs Mn^4+^/Mn^2+^ can scavenge the overexpressed GSH and catalyze H_2_O_2_ decomposition to generate O_2_.^[^ ^7^ ^]^ Therefore, the Mn^2+^‐containing nanocomposite is a prominent candidate for achieving ICD and TME‐responsive CDT.

Starvation therapy is another new and non‐invasive oncotherapy strategy based on glucose oxidase (GOx)‐mediated catalysis, which has attracted increasing attention owing to its negligible side effects.^[^ ^8^ ^]^ GOx catalyzes the conversion of glucose into gluconic acid and H_2_O_2_, thereby limiting the essential energy availability and ultimately suppressing tumor proliferation and metastasis.^[^ ^9^ ^]^ Previous studies have shown that the combination of GOx and CDT is an effective strategy for killing tumors.^[^ ^10^ ^]^ Shi et al. constructed a sequential nanocatalyst wherein dendritic mesoporous silica nanoparticles (DMSNs) were used as nanocarriers to co‐deliver GOx and ferroferric oxide (Fe_3_O_4_) nanoparticles. GOx catalyzes the glucose to produce H_2_O_2_, facilitating the generation of •OH by Fe_3_O_4_ for CDT.^[^ ^11^ ^]^ However, the hypoxic condition of TME and the low biodegradation efficiency of DMSNs severely limit the catalytic performance of GOx. Compared with the DMSNs, the disulfide‐bond‐incorporated dendritic mesoporous organosilica nanoparticles (DMONs) with a central‐radial pore structure and highly accessible surface areas are considered to be ideal nanocarriers for therapeutic molecule delivery.^[^ ^12^ ^]^ Based on the GSH‐responsive disulfide bonds, it can be degraded and released the inner cargo to achieve the desired effects. In addition, compared with other widely used metal–organic frameworks or covalent‐organic frameworks, such as ZIF‐8, ZIF‐67, and COF‐909,^[^ ^13^ ^]^ DMONs hold great promise for more broad application prospects due to the high drug loading efficiency, low toxicity, and strong biodegradation efficiency. Thus, the design of appropriate nanocarriers based on DMONs to explore more efficient catalytic cascade strategies and alteration of the special conditions of the TME are imperative.

In addition to chemodynamic and starvation therapies, immunotherapy is another non‐invasive strategy that mobilizes immune cells to attack tumors.^[^ ^14^ ^]^ Although the combination of CDT and starvation therapy can effectively induce ICD, the immunosuppressive TME restricts its effectiveness.^[^ ^15^ ^]^ Among the plenty of immunosuppressive mechanisms, the upregulated immune checkpoint protein indoleamine 2,3‐dioxygenase (IDO) can inhibit the activity of effector T cells and increase the number of regulatory T (Treg) cells by catabolizing tryptophan (Trp) to kynurenine (Kyn).^[^ ^16^ ^]^ Trp is one of the main nutrients for lymphocytes, whereas toxic Kyn can induce lymphocyte apoptosis. Moreover, depleted Trp can incapacitate lymphocytes, rendering them incapable of attacking cancer cells. However, IDO inhibitors can inhibit IDO to reduce Trp catabolism and restore T lymphocyte activity. Therefore, the use of IDO inhibitors is an effective method to destroy the barrier of negative immunosuppressive mechanisms. Li et al. used a self‐delivering oxidative stress amplifier that utilized the self‐assembly effect of copper ions (Cu^2+^), doxorubicin (DOX), and NLG919 to achieve chemodynamic/chemotherapy‐sensitized immunotherapy. NLG919 reversed tumor immunosuppressive microenvironment to enhance ICD induced by Cu^2+^‐activated GSH consumption and DOX‐mediated oxidative stress.^[^ ^3c^ ^]^ However, the strong side effects of DOX and weak cascade effects restrict the application of nanocomposites. Accordingly, synergistic combination therapy nanocomposites that combine IDO inhibitors and other therapy methods have been preliminarily established, although more reasonable strategies with fewer side effects still need to be developed. Therefore, the integration of chemodynamic/starvation therapy and IDO inhibitors into a single nanocomposite may help overcome the challenges of monotherapy.

**Scheme 1:**
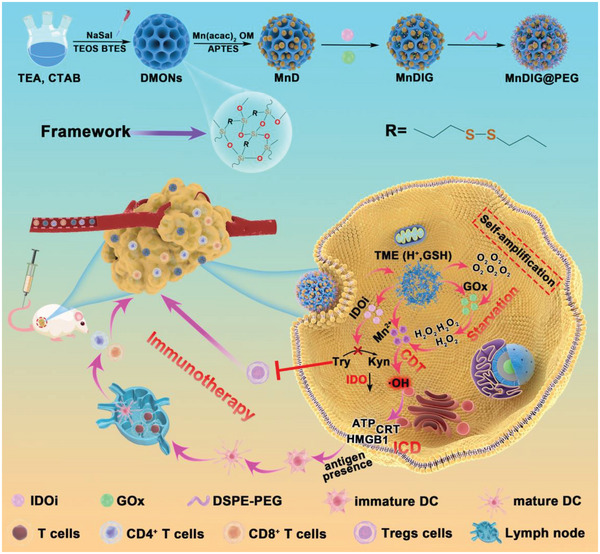
Schematic illustration of the synthesis and antitumor mechanism of the MnDIG@PEG nanocomposite. Here, the large‐pore organosilica mesoporous‐silica decorated Mn_3_O_4_ nanoparticles as the cornerstone are prepared first. Then by loading IDOi, GOx, and modifying the DSPE‐PEG. After injecting the obtained MnDIG@PEG nanocomposite into tumor bearing BALB/c mice, the nanocomposites can rapidly decompose in the endogenous TME, release Mn^2+^, GOx, and IDOi. The self‐amplification chemodynamic/starvation therapy can enhance immunogenic cell death, while IDOi inhibit the IDO activity. Taking advantage of enhanced immune response by Mn^2+^/GOx‐mediated chemodynamic/starvation therapy and immune suppression performed by IDO blockade immunotherapy, the MnDIG@PEG nanocomposite can achieve a conspicuous therapeutic effect.

Based on the above considerations, we constructed a TME‐responsive nanocomposite (MnDIG@PEG) using manganese tetroxide (Mn_3_O_4_)‐decorated DMONs to co‐deliver the IDO inhibitor Epacadostat (IDOi) and GOx following by modification with polyethylene glycol (PEG) for chemodynamic/starvation therapy enhanced IDO‐blockade immunotherapy, as shown in **Scheme** **1**. The obtained MnDIG@PEG nanocomposite has the following merits: a) the large pore structure of the DMONs provides a high drug loading efficiency; b) the synthesized MnDIG@PEG nanocomposite underwent rapid degradation in the mildly acidic and GSH overexpressed TME, improving its biocompatibility and release efficiency; c) the consumption of glucose by GOx is accompanied by increased H_2_O_2_ production, which can be further utilized by Mn^2+^ to generate highly cytotoxic •OH, leading to ICD. Moreover, IDOi can inhibit IDO activity, thereby facilitating self‐amplifying chemodynamic/starvation therapy combined with IDO‐blockade immunotherapy. The MnDIG@PEG nanocomposite exhibited good therapeutic capacity as a result of self‐amplifying chemodynamic/starvation therapy and alleviation of the immune‐suppressive microenvironment by IDO‐blockade immunotherapy. Therefore, the successfully constructed MnDIG@PEG nanocomposite provides a platform for improving antitumor immunity and inhibiting tumor growth and metastasis through TME‐responsive self‐amplifying oncotherapeutic strategies.

## 2 Results and Discussion

**Figure 1:**
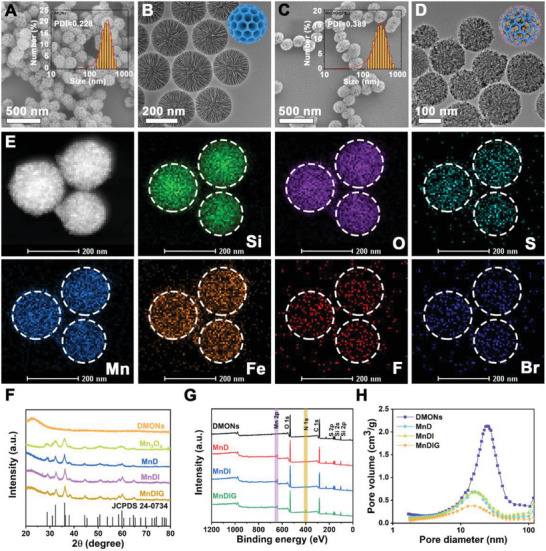
SEM images of A) DMONs, and C) MnDIG@PEG (inset: hydrodynamic diameters by DLS measurement). TEM images of B) DMONs, and D) MnDIG@PEG. E) The High‐angle annular dark‐field scanning TEM (HAADF‐STEM) image of MnDIG@PEG and the corresponding elemental mappings, including Si, O, S, Mn, Fe, F, and Br. F) XRD patterns, G) full XPS spectra, and H) pore size distribution of various formulations.

Here, DMONs were initially synthesized as previously described,^[^ ^17^ ^]^ which showed a dendritic spherical structure with a diameter of approximately 200 nm (**Figure** **1**A,B). Subsequently, Mn_3_O_4_ nanoparticles were introduced in situ onto the surface of the DMONs to obtain Mn_3_O_4_@DMONs (MnD) nanocomposites (Figure S1, Supporting Information). Mn_3_O_4_ exhibited a discrete spherical morphology with a diameter of approximately 6 nm, and high‐resolution transmission electron microscopy (TEM) and selected‐area electron‐diffraction confirmed that it was highly crystalline (Figure S2, Supporting Information). Subsequently, amine groups were selected to replace the oleylamine groups of Mn_3_O_4_ and modify the DMONs. The morphology and size of MnDIG@PEG barely changed after loading IDOi, GOx, and modifying DSPE‐PEG (Figure 1C,D; Figure S3, Supporting Information). The zeta potential variations of the obtained samples also demonstrated that the MnDIG@PEG nanocomposite was successfully prepared (Figure S4, Supporting Information). The final zeta potential and average hydrodynamic particle size of MnDIG@PEG were −8.3 mV and 318 nm, respectively. Additionally, the constructed MnDIG@PEG exhibited good stability in PBS, RPMI‐1640, and fetal bovine serum (Figure S5, Supporting Information). High‐angle annular dark‐field scanning TEM and elemental mapping images showed that all elements (Si, O, S, Mn, Fe, F, and Br) were homogeneously distributed, revealing the coexistence of the different elements in the MnDIG@PEG nanocomposite (Figure 1E). Furthermore, the color difference between the DMONs and MnDIG@PEG confirmed the successful synthesis of the final nanocomposite (Figure S6, Supporting Information). The X‐ray diffraction patterns of the different samples are shown in Figure 1F, the characteristic diffraction peaks of MnDIG@PEG indicate the presence of Mn_3_O_4_. In addition, the contact angle of MnDIG was above 90°, and it was decreased obviously after modifying with DSPE‐PEG, which confirmed the successful modification of the DSPE‐PEG (Figure S7, Supporting Information). Meanwhile, as shown in Figure S8A (Supporting Information), the mass change at each preparation stage demonstrated the successful construction of the MnDIG@PEG nanocomposite. Moreover, new peaks belonging to IDOi, GOx, and DSPE‐PEG appeared in the MnDIG@PEG nanocomposite (Figure S8B, Supporting Information), further confirming the successful construction of the MnDIG@PEG nanocomposite.

To further confirm the successful construction of the MnDIG@PEG nanocomposite, the X‐ray photoelectron spectroscopy spectra of different samples were measured. As exhibited in Figure S9A,B (Supporting Information), the existence of Si 2p and S 2p peaks confirmed the successful preparation of disulfide‐bond‐incorporated DMONs. Subsequently, compared with DMONs, the emerging peaks of Mn 2p and N 1s were observed in Mn_3_O_4_@DMONs‐IDOi‐GOx (MnDIG), confirming the successful decoration of Mn_3_O_4_ and amine groups (Figure 1G; Figure S9C,D, Supporting Information). In addition, as shown in Figure 1H and Figure S10 (Supporting Information), the N_2_ adsorption–desorption isotherms and the corresponding pore size distributions of the different samples were determined. After decorating with Mn_3_O_4_, the specific surface area of MnD (217.5 m^2^ g^−1^) was reduced compared with that of DMONs (382.5 m^2^ g^−1^), and the corresponding pore size (approximately 11 nm) was also changed. Moreover, the specific surface area and pore size were further reduced upon loading of IDOi and GOx and modification with DSPE‐PEG. Thus, all the results demonstrated the successful construction of the MnDIG@PEG nanocomposite.

**Figure 2:**
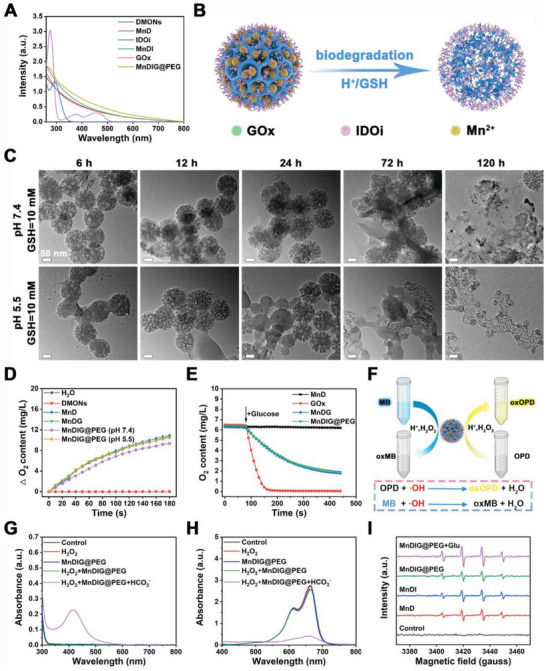
A) UV–vis absorption spectra of different samples. B) Schematic illustration of pH/GSH‐triggered biodegradation process of MnDIG@PEG. C) TEM images of MnDIG@PEG after biodegradation treated under different conditions. D) O_2_ generation profiles of different samples. E) O_2_ consumption profiles of different samples in the presence of glucose. F) Schematic illustration of the catalytic procedure of MnDIG@PEG. UV–vis absorption spectra of the G) OPD and H) MB incubated with different samples. I) ESR spectra of DMPO mixed with different conditions for 30 min.

The most important characteristics of the MnDIG@PEG nanocomposite are cargo delivery and TME‐responsive biodegradation capacity, which are conducive to improving self‐amplifying chemodynamic/starvation therapy combined IDO‐blockade immunotherapy. Prior to the responsive measurements, the loading efficiencies of IDOi and GOx were determined. As displayed in **Figure** **2**A, the UV–vis absorption spectrum of the obtained samples was highly consistent. Subsequently, we evaluated the loading efficiency using high‐performance liquid chromatography and UV–vis absorption spectroscopy. The concentration of IDOi in the Mn_3_O_4_@DMONs‐IDOi (MnDI) supernatant was measured, and the loading efficiency was found to be 2.7% (Figure S11, Supporting Information). Similarly, we measured the concentration of GOx in the MnDIG supernatant and calculated the loading efficiency of GOx to be 20.8% (Figure S12, Supporting Information). These results indicate that MnD can be used as a nanocarrier to improve the cargo delivery capacity.

Because of the sensitivity of the disulfide‐bond‐incorporated DMONs and Mn_3_O_4_ to H^+^/GSH, we further investigated the TME‐responsive biodegradation and cargo release capacity of the MnDIG@PEG nanocomposite (Figure 2B). Figure 2C shows the TEM images of MnDIG@PEG under different conditions. In the presence of GSH, the structural destruction of MnDIG@PEG was evident in both neutral and mildly acidic environments as time increased from 6 to 120 h. However, the structure integrity collapsed more noticeably in the mildly acidic environment. After 120 h, the MnDIG@PEG nanocomposite had almost completely decomposed, leading to the release of Mn ions and cargo. In addition, the color and absorbance of the MnDIG@PEG solution weakened as the concentration of GSH increased (Figure S13, Supporting Information), thus confirming TME‐responsive biodegradation. Subsequently, we investigated the delivery behaviors of the different cargoes. As exhibited in Figure S14A (Supporting Information), an inappreciable Mn ions release was detected by inductively coupled plasma‐mass spectrometry at pH 7.4 or 6.5, indicating the high stability of the nanocomposite. In contrast, Mn ions were released more rapidly with decreasing pH and GSH addition, confirming the TME‐responsive cargo release capacity. Similarly, GOx and IDOi showed the highest release in acidic GSH solution compared with other conditions (Figure S14B,C, Supporting Information). This characteristic ensures efficient cargo transport to the tumor and avoids causing side effects to normal tissue.

Based on the TME‐responsive biodegradation of the MnDIG@PEG nanocomposite, we explored the catalytic activities of Mn_3_O_4_ and GOx. Titanic sulfate [Ti(SO_4_)_2_] is generally used as an indicator to evaluate the consumption of H_2_O_2_, as indicated when its color changes from yellow to colorless.^[^ ^18^ ^]^ As displayed in Figure S15A (Supporting Information), owing to the catalytic capacity of Mn_3_O_4_, the Mn_3_O_4_‐decorated nanocomposites exhibited rapid H_2_O_2_ consumption over 30 min, according to the standard curve of absorbance versus the concentration of H_2_O_2_ (Figure S15B,C, Supporting Information). Moreover, the average hydrodynamic particle size of MnDIG@PEG changed after incubation with acid H_2_O_2_, which confirmed the decomposition of Mn_3_O_4_ (Figure S15D, Supporting Information). In addition, Mn_3_O_4_ catalyzed the decomposition of H_2_O_2_ to generate O_2_ via the following reaction:

(1) $$2H_{2}O_{2}\overset{{\mathrm{Mn}}_{3}O_{4}}{\to }2H_{2}O+O_{2}$$

As exhibited in Figure 2D, compared with the control group (H_2_O and DMONs), the production of O_2_ catalyzed by the Mn_3_O_4_‐decorated nanocomposite increased in a time‐dependent manner upon incubation with H_2_O_2_, and acid environment could also accelerate the catalysis process. Furthermore, the catalytic activities of free GOx and MnDIG@PEG were detected in the presence of glucose, which manifested as the consumption of glucose and O_2_ and a change in pH.^[^ ^19^ ^]^ As shown in Figure S15E (Supporting Information), compared with the control group, the glucose concentration in other groups (GOx, MnDG, and MnDIG@PEG) gradually decreased during the catalytic process. Owing to the protective effects of the DMONs, the catalytic efficiency of MnDIG@PEG slightly decreased. Furthermore, the change in dissolved O_2_ content and pH values also demonstrated the same outcomes (Figure 2E; Figure S15F, Supporting Information). Moreover, the biological activity of GOx in simulated in vivo release environments is shown in Figure S16 (Supporting Information). Compared with the free GOx, the catalytic activity of MnDIG@PEG was little decreased over 6 h; moreover, the biological activity of free GOx decreased more than that of MnDIG@PEG over 48 h, which was attributed to the protective effects of the DMONs. These results confirmed the desirable catalytic capacity of the MnDIG@PEG nanocomposite, which makes it suitable for further applications.

Self‐amplifying •OH generation was evaluated based on the catalytic capacity of the MnDIG@PEG nanocomposites. Typically, o‐phenylenediamine (OPD) and methylene blue (MB) are the common indicators used for assessing •OH production, and show absorbance at 437 and 664 nm, respectively (Figure 2F). Unlike other Fenton reagents, bicarbonate (HCO_3_^−^) is indispensable for Mn ions‐activated Fenton‐like reactions. Thus, as demonstrated in Figure 2G,H, and S17 (Supporting Information), only MnDIG@PEG catalyzed H_2_O_2_ to produce •OH with the help of HCO_3_^−^, and the catalysis process was affected by pH values. Furthermore, as the concentration of H_2_O_2_ increased, the absorbance of OPD increased and that of MB decreased, revealing that the production of •OH depended on the amount of H_2_O_2_ (Figure S18A,B, Supporting Information). To further prove •OH generation, electron‐spin‐resonance spectra were employed using 5,5‐dimethyl‐1‐pyrroline‐N‐oxide (DMPO) as a trapper. As shown in Figure 2I, the unique •OH signal was observed after treatment under the aforementioned conditions. The •OH signal of MnDIG@PEG was enhanced after the addition of glucose, indicating that GOx catalyzed the generation of H_2_O_2_ from glucose and promoted •OH production. Moreover, the change in the absorbance of OPD and MB with increasing glucose also demonstrated the capacity of GOx to enhance •OH generation (Figure S18C,D, Supporting Information). It is worth noting that the overexpressed GSH as a reactive oxygen species scavenger in tumors also hinders the action of •OH; therefore, the depletion of GSH by MnDIG@PEG was further measured. 5,5′‐dithiobis‐(2‐nitrobenzoic acid) (DTNB), which reacts with GSH to form a yellow‐colored solution with an absorption peak at 412 nm, was applied to assess the consumption of GSH. As shown in Figure S19A (Supporting Information), the absorbance of DTNB at 412 nm only increased when the concentration of GSH exceeded 4 mm, demonstrating the depletion of GSH by MnDIG@PEG. Furthermore, the degradation of MB and oxidation of OPD increased with elevating GSH concentration but decreased substantially as GSH increased, which also confirmed the GSH‐depleting effects of the nanocomposite (Figure S19B,C, Supporting Information). The results proved that the MnDIG@PEG nanocomposite not only generated H_2_O_2_ but also eliminated overexpressed GSH to achieve self‐amplifying CDT.

**Figure 3:**
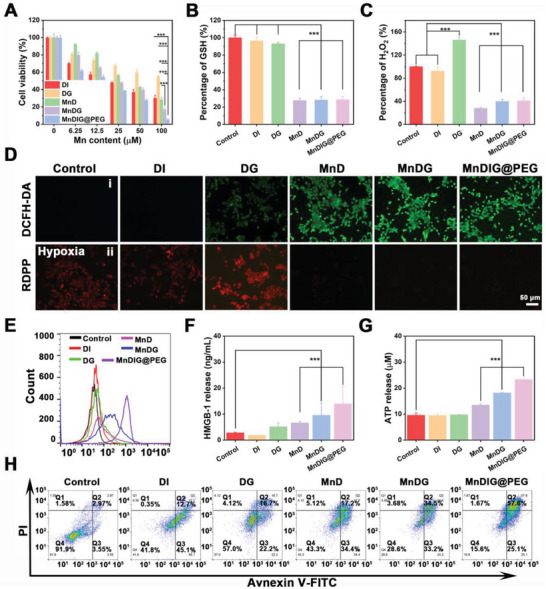
A) Cell survival rates of 4T1 cells incubation for 24 h after different treatments. B) Intracellular GSH detections of 4T1 cells after 6 h of incubation with different samples. C) Intracellular H_2_O_2_ detections of 4T1 cells after 6 h of incubation with different samples. D) The detection of i) ROS or ii) O_2_ production in 4T1 cells by staining with DCFH‐DA or RDPP after different treatments. E) The CRT exposure in 4T1 cells after different treatments. F) The HMGB1 release in 4T1 cells after different treatments. G) The ATP release in 4T1 cells after different treatments. H) Flow cytometry apoptosis assay of 4T1 cells after different treatments for 24 h followed by staining with Annexin‐FITC and PI (n = 3, mean ± SD; ^*^ *p* <0.05; ^**^ *p* <0.01; ^***^ *p* <0.001).

After confirming the successful construction and effects of the MnDIG@PEG nanocomposite, we further explored its application in tumor treatment both in vitro and in vivo. As biocompatibility of nanocomposites is an important requirement in tumor therapy, the MTT assay was employed to assess the cytotoxicity of the nanocomposite against mouse fibroblasts (L929) cells, Dendritic (DC 2.4) cells, and mouse breast cancer (4T1) cells. As shown in Figure S20A (Supporting Information), the L929 cells always showed a low death rate even at an Mn concentration of 100 µM in MnD, suggesting the favorable biocompatibility of the MnD nanocarrier. Furthermore, MnDIG@PEG also exhibited lower cytotoxicity against L929 cells, DC cells, and NIH‐3T3 cells (Figure S20B,D, Supporting Information). As displayed in **Figure** **3**A, DMONs‐GOx (DG) exhibited starvation therapy damage of 38.56 µm IC_50_, and MnD showed CDT injury of 40.99 µm IC_50_. Therefore, the cooperative index (CI) was calculated to be 0.62 (CI <0.8, considered a significant cooperative effect),^[^ ^20^ ^]^ indicating the synergistic effect of CDT and starvation therapy. In addition, fairly low 4T1 cell viability was observed after treatment with MnDIG@PEG compared with other groups, which was induced by the high efficiency of the combined therapies. Moreover, MnDIG@PEG also exhibited higher cytotoxicity against Hela cells (Figure S21, Supporting Information). Subsequently, the cellular endocytosis and intracellular distribution of MnDIG@PEG were explored via fluorescence imaging and ICP‐MS. For this purpose, MnDIG@PEG was first marked by Rhodamine B (RB). As shown in Figure S22 (Supporting Information), the typical red fluorescence of MnDIG@PEG‐RB was widely distributed over the 4T1 cells as the incubation time increased from 0.5 to 6 h; moreover, the intracellular Mn content improved as the cultivation time increased (Figure S23, Supporting Information suggesting good tumor internalization of the nanocomposite. More visually, the bio‐TEM images showed that 4T1 cells treated with MnDIG@PEG at different times exhibited time‐dependent internalization and structural damage owing to mild acid and GSH responses, as shown in Figure S24 (Supporting Information). In addition, a distinct red fluorescence signal and separation of red and green dots were observed in the cytoplasm after incubation with MnDIG@PEG from 0.5 to 12 h, suggesting a certain degree of endo/lysosome escape (Figure S25, Supporting Information). The endo/lysosomal escape ability of MnDIG@PEG was very likely due to the reduced lysosomal membrane stability.^[^ ^21^ ^]^

Considering the GSH depletion and H_2_O_2_ consumption by MnDIG@PEG, intracellular GSH and H_2_O_2_ levels were investigated. As displayed in Figure 3B, according to the standard curve of GSH (Figure S26A, Supporting Information), the GSH content of 4T1 cells was slightly attenuated after treatment with DMONs‐IDOi (DI) and DG, which was attributed to the elimination of GSH by the disulfide‐bond‐incorporated DMONs. However, after modification with Mn_3_O_4_, the resulting nanocomposites rapidly consumed GSH, which aided in disrupting intracellular redox homeostasis and improving the efficiency of CDT. Moreover, as presented in Figure 3C, compared with the control and DI groups, DG substantially increased the H_2_O_2_ level according to the standard curve of absorbance versus concentration of H_2_O_2_ (Figure S26B, Supporting Information), which was attributed to the GOx‐induced catalytic effect on glucose. In contrast, Mn_3_O_4_ rapidly consumed H_2_O_2_ to generate O_2_ and •OH; thus, the H_2_O_2_ levels of other groups decreased sharply, implying that these nanocomposites eliminated the overexpressed GSH and consumed H_2_O_2_. Subsequently, fluorescence imaging of 2′,7′‐dichlorofluorescein generated from oxidation of 2′,7′‐dichlorodihydrofluorescein diacetate by ROS was employed to accurately evaluate the production of •OH (Figure 3D). Compared with the control and DI groups, weak green fluorescence was observed in the DG group because GOx could increase the level of H_2_O_2_ to disrupt intracellular redox homeostasis. In addition, a stronger green fluorescence was observed in the MnD group, which was attributed to the production of •OH by the Mn‐based Fenton‐like reaction. Furthermore, with the assistance of GOx, the green fluorescence was obviously improved in both MnDG and MnDIG@PEG groups. And the green fluorescence was further enhanced by the addition of glucose, indicating the action of self‐amplifying chemodynamic/starvation therapy (Figure S27, Supporting Information). Owing to the GSH depletion and •OH generation, MnDIG@PEG exhibited an obvious concentration‐dependent lipid peroxidation rise, indicating the occurrence of ferroptosis (Figure S28, Supporting Information). Moreover, intracellular glucose and O_2_ levels were measured to further analyze the catalytic activity of GOx. Compared with other groups, the groups loaded with GOx consumed intracellular glucose; moreover, the catalytic activity was influenced slightly after modifying with DSPE‐PEG (Figure S29, Supporting Information). The results of O_2_ fluorescence imaging under hypoxia are shown in Figure 3D; the stronger red fluorescence in the DG group than that of the control group indicated the consumption of O_2_ by GOx. However, the fluorescence signals largely disappeared in the MnD, MnDG, and MnDIG@PEG groups, further proving the production of O_2_ after decoration with Mn_3_O_4_. Along with the catalytic reaction duration, the •OH production was also improved, which further induced endosomal membrane oxidation and rupture, as well as mitochondrial damage (Figures S30–S32, Supporting Information).

Notably, it should be known that CDT can trigger ICD by releasing damage‐associated molecular patterns as in previous reports.^[^ ^22^ ^]^ Subsequently, crucial ICD biomarkers were measured to accurately determine whether the MnDIG@PEG nanocomposite could stimulate ICD. As displayed in Figure 3E, compared with other groups, the highest level of cell surface calreticulin (CRT) exposure was detected in the MnDIG@PEG group. Additionally, significant high‐mobility group box 1 (HMGB1) release (Figure 3F) and adenosine triphosphate secretion (Figure 3G) were observed under the corresponding conditions, confirming the capacity of the MnDIG@PEG nanocomposite to elicit ICD via GOx assisted CDT. Subsequently, flow cytometry analysis was employed to analyze the cell apoptosis ratio of the 4T1 cells after incubation with different samples. As shown in Figure 3H, owing to the loading of IDOi and GOx, the MnDIG@PEG nanocomposite resulted in a higher apoptosis ratio of 4T1 cells (82.7%) compared with other groups due to self‐amplifying chemodynamic/starvation therapy. IDO catalyzes the metabolism of Trp to Kyn, contributing to an immunosuppressive TME that restricts the efficiency of therapeutic agents. Thus, IDO enzyme activity was explored by measuring the conversion of Trp to Kyn after treatment with different samples. As shown in Figure S33 (Supporting Information), the MnDIG@PEG nanocomposite inhibited the IDO enzyme activity compared with other groups, as indicated by the inhibition rate of Kyn. According to the above results, the superior antitumor effect of the constructed nanocomposite in vitro owing to the self‐amplifying combined therapies of MnDIG@PEG, which was further proven by subsequent in vivo experiments.

**Figure 4:**
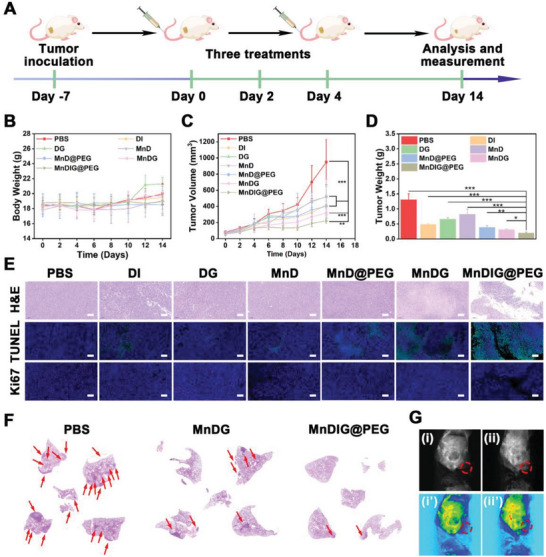
A) Schematic description of the establishment of 4T1 xenograft tumor and therapeutic outcome with MnDIG@PEG. B) Body‐weight, C) tumor‐volume, and D) tumor weight of tumor‐bearing mice with different treatments. E) H&E, TUNEL, and Ki67 staining images for tumors. Scale bars: 100 µm. F) H&E staining of lungs in anti‐metastatic studies. G) In vivo *T*_1_‐weighted MRI of i,i’) PBS and ii,ii’) MnDIG@PEG (n = 5, mean ± SD; ^*^ *p* <0.05; ^**^ *p* < 0.01; ^***^ *p* <0.001).

To further validate the superiority of the combined therapies, the in vivo antitumor efficiency was measured in the xenograft 4T1 tumor model. As displayed in **Figure** **4**A, the xenograft 4T1 tumor was established on the right back of BALB/c mice to investigate the antitumor and antimetastatic effects of the MnDIG@PEG nanocomposite. First, the in vivo biocompatibility of MnDIG@PEG was measured methodically. As illustrated in Figure S34 (Supporting Information), no obvious inflammation or organ damage was monitored in H&E staining images after treatment with MnDIG@PEG for 0, 7, 14, and 30 days. In addition, the in vivo blood biochemistry, including whole blood and serum analyses revealed no abnormal manifestations between the control and MnDIG@PEG groups within 30 days (Figure S35, Supporting Information). Furthermore, no noticeable differences were observed in the body weights of the mice treated with various nanocomposites throughout the observation period (Figure 4B). After intravenous injection, the blood half‐life of the MnDIG@PEG nanocomposite was measured to be 1.53 h (Figure S36A, Supporting Information). Moreover, the bio‐distribution of the MnDIG@PEG nanocomposites in major organs and tumors was determined simultaneously. As shown in Figure S36B (Supporting Information), the corresponding Mn ions content at the tumor site was up to 3.8% after intravenous injection for 1 h and increased to 6.5% at 2 h, demonstrating that the MnDIG@PEG nanocomposite could be enriched at the tumor site to eliminate tumor cells. The results of in vivo fluorescence imaging also proved the accumulation of MnDIG@PEG nanocomposite in tumor sites (Figure S37, Supporting Information). The excretion of the MnDIG@PEG nanocomposites was also evaluated. As shown in Figure S38 (Supporting Information), Mn was rapidly excreted from the mice within 24 h of administration, indicating that MnDIG@PEG was easily degraded and excreted from the body. Owing to the high biocompatibility and tumor enrichment capacity of the MnDIG@PEG nanocomposite, we further investigated the synergistic antitumor effects. All the tumor‐bearing mice were randomly divided into seven groups (n = 5): (1) control; (2) DI; (3) DG; (4) MnD; (5) MnD@PEG; (6) MnDG; (7) MnDIG@PEG. After intravenous injection of the different nanocomposites for three times, the corresponding tumor volume and weight were measured throughout the experimental period. Compared with the control group, the DG, DI, and MnD groups exhibited similar tumor growth inhibition rates, indicating that the single therapeutic schedule had a weak antitumor effect. However, modification with DSPE‐PEG improved the therapeutic effects of MnD. In addition, the MnDG group exhibited moderate tumor growth inhibition, which was attributed to the combination of chemodynamic and starvation therapies. In comparison, tumor growth inhibition was most distinct in the MnDIG@PEG group, indicating that self‐amplifying chemodynamic/starvation therapy combined with IDO‐blockade immunotherapy could lead to the most effective oncotherapy (Figure 4C). In addition, the results of tumor weight (Figure 4D), digital photographs of tumor‐bearing mice (Figure S39, Supporting Information), and extracted tumors (Figure S40, Supporting Information) further confirmed this. Additionally, H&E staining, Terminal Deoxynucleotidyl Transferase‐mediated dUTP Nick‐End Labeling (TUNEL), and Ki67 staining were performed to systematically demonstrate the antitumor efficiency of the MnDIG@PEG nanocomposite. As shown in Figure 4E, MnDIG@PEG resulted in severe tumor damage, as demonstrated by noticeable tissue destruction in the H&E staining results, numerous green dots in the TUNEL images, and weakened Ki67 expression. Moreover, hypoxia‐inducible factor‐1α staining images proved that the nanocomposite could significantly ameliorate tumor hypoxia (Figure S41, Supporting Information). Simultaneously, the CRT, HMGB1, and IDO staining images proved that the nanocomposite induced ICD and inhibited IDO expression to enhance the immune response (Figure S42, Supporting Information). In addition, the H&E images of major organs with different treatments after 14 days showed that MnDIG@PEG had negligible adverse effects on normal tissues during therapy (Figure S43, Supporting Information).

The noninvasive MRI was used to evaluate the accumulation of the MnDIG@PEG nanocomposite. As illustrated in Figure S44A (Supporting Information), the in vitro results indicated that the r_1_ value was 4.246 mm ^−1^ s^−1^ after the administration of the MnDIG@PEG nanocomposite with acidic GSH, and the MRI intensity was accordingly enhanced (Figure S44B, Supporting Information). Moreover, compared with the PBS group, the MnDIG@PEG group showed a distinct *T_1_*‐weighted signal at the tumor site (Figure 4G). These results demonstrated that MnDIG@PEG can be utilized for TME‐responsive MRI to monitor the accumulation of nanocomposites.

**Figure 5:**
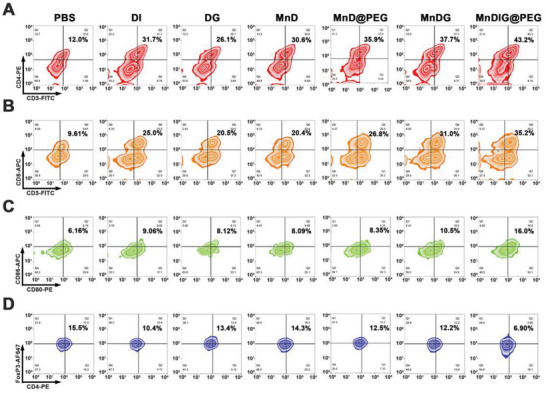
Flow cytometric analyses of the populations of A) CD4^+^ T cells, B) CD8^+^ T cells, C) DC cells, and D) Treg cells collected from spleens of tumor‐bearing mice with different treatments.

Encouraged by the tumor suppressive efficiency and activated ICD effects of the MnDIG@PEG nanocomposite, the antimetastatic effect and antitumor immune response of the nanocomposite were further evaluated. Lung metastasis was monitored using H&E stained images of lung tissues extracted from tumor‐bearing mice after different treatments for 28 days. As shown in Figure 4F and Figure S45 (Supporting Information), almost no lung nodules were observed in the MnDIG@PEG group, indicating that MnDIG@PEG nanocomposite had desirable antimetastatic effects. Furthermore, interrelated immune cells in spleens and tumors of tumor‐bearing mice were quantitatively analyzed by flow cytometry. As shown in **Figure** **5** and Figures S46–S49 (Supporting Information), a small fraction of cytotoxic T cells (Figure 5A,B), and mature DC cells (Figure 5C), as well as a large number of Treg cells (Figure 5D) were observed in the control group, which was attributed to the innate immunosuppressive microenvironment. Simultaneously, owing to the inhibition of IDO1 by IDOi, a notable increase in the number of cytotoxic T cells and mature DC cells was observed in the DI group whereas Treg cells exhibited a moderate decrease. In addition, the DG, MnD, MnD@PEG, and MnDG groups also exhibited a moderate performance in stimulating the antitumor immunity, but had almost no effect on the inhibition of Treg cell proliferation. Remarkably, MnDIG@PEG induced substantial cytotoxic T cell, mature DC cells proliferation, and weakest Treg cell proliferation, indicating a strengthened antitumor immune response due to a less immunosuppressive microenvironment. Therefore, the multifunctional TME‐responsive MnDIG@PEG nanocomposite synergistically eliminated tumor cells and inhibited tumor metastasis through self‐amplifying chemodynamic/starvation therapy and alleviation of the immunosuppressive microenvironment via IDO‐blockade immunotherapy.

## 3 Conclusion

In this study, a multifunctional TME‐responsive nanocomposite was constructed with an enhanced release ability to co‐deliver IDOi and GOx for self‐amplifying chemodynamic/starvation therapy combined with IDO‐blockade immunotherapy. The obtained in vitro and in vivo results demonstrated that the MnDIG@PEG nanocomposite can effectively improve delivery efficiency via its large pore structure and TME‐responsive ability as well as enhance antitumor immunity by inducing ICD and IDO‐blockade immunotherapy. Owing to the advantages of these strategies, the MnDIG@PEG nanocomposite efficiently inhibited tumor growth and metastasis. In summary, the successfully constructed MnDIG@PEG nanocomposite provides a platform for improving antitumor immunity to inhibit tumor growth and metastasis through TME‐responsive self‐amplifying multimodal therapeutic strategies for oncotherapy.

## Conflict of Interest

The authors declare no conflict of interest.

## Supporting information

Supporting Information

## Data Availability

The data that support the findings of this study are available from the corresponding author upon reasonable request.

## References

[1] a) Q. Liu , Y. J. Kim , G. B. Im , J. Zhu , Y. Wu , Y. Liu , S. H. Bhang , Adv. Funct. Mater. 2020, 31, 2008171;

[2] a) Z. Tang , Y. Liu , M. He , W. Bu , Angew. Chem., Int. Ed. 2019, 58, 946; 10.1002/anie.201805664 30048028

[3] a) B. Ding , P. Zheng , F. Jiang , Y. Zhao , M. Wang , M. Chang , P. Ma , J. Lin , Angew. Chem., Int. Ed. 2020, 59, 16381; 10.1002/anie.202005111 32484598

[4] a) L. S. Lin , J. Song , L. Song , K. Ke , Y. Liu , Z. Zhou , Z. Shen , J. Li , Z. Yang , W. Tang , G. Niu , H. H. Yang , X. Chen , Angew. Chem., Int. Ed. 2018, 57, 4902; 10.1002/anie.201712027 29488312

[5] A. D. Bokare , W. Choi , J. Hazard. Mater. 2014, 275, 121. 24857896 10.1016/j.jhazmat.2014.04.054

[6] B. Ding , S. Shao , F. Jiang , P. Dang , C. Sun , S. Huang , P. a. Ma , D. Jin , A. A. A. Kheraif , J. Lin , Chem. Mater. 2019, 31, 2651.

[7] a) B. Xu , Y. Cui , W. Wang , S. Li , C. Lyu , S. Wang , W. Bao , H. Wang , M. Qin , Z. Liu , W. Wei , H. Liu , Adv. Mater. 2020, 32, 2003563; 10.1002/adma.202003563 32627937

[8] L. Dai , M. Yao , Z. Fu , X. Li , X. Zheng , S. Meng , Z. Yuan , K. Cai , H. Yang , Y. Zhao , Nat. Commun. 2022, 13, 2688. 35577812 10.1038/s41467-022-30436-y PMC9110376

[9] M. Wang , M. Chang , C. Li , Q. Chen , Z. Hou , B. Xing , J. Lin , Adv. Mater. 2022, 34, 2106010. 10.1002/adma.202106010 34699627

[10] X. Zhang , C. He , Y. Chen , C. Chen , R. Yan , T. Fan , Y. Gai , T. Yang , Y. Lu , G. Xiang , Biomaterials 2021, 275, 120987. 34175561 10.1016/j.biomaterials.2021.120987

[11] M. Huo , L. Wang , Y. Chen , J. Shi , Nat. Commun. 2017, 8, 357. 28842577 10.1038/s41467-017-00424-8 PMC5572465

[12] a) Z. Guo , H. He , Y. Zhang , J. Rao , T. Yang , T. Li , L. Wang , M. Shi , M. Wang , S. Qiu , X. Song , H. Ke , H. Chen , Adv. Mater. 2021, 33, 2004225; 10.1002/adma.202004225 33270303

[13] a) S. Gao , Y. Jin , K. Ge , Z. Li , H. Liu , X. Dai , Y. Zhang , S. Chen , X. Liang , J. Zhang , Adv. Sci. 2019, 6, 1902137; 10.1002/advs.201902137 PMC6918120 31871871

[14] M. Saeed , F. Chen , J. Ye , Y. Shi , T. Lammers , B. G. De Geest , Z. P. Xu , H. Yu , Adv. Mater. 2021, 33, 2008094. 10.1002/adma.202008094 34048101

[15] J. Guan , Y. Wu , X. Liu , H. Wang , N. Ye , Z. Li , C. Xiao , Z. Zhang , Z. Li , X. Yang , Biomaterials 2021, 279, 121180. 34768152 10.1016/j.biomaterials.2021.121180

[16] Y. Lu , F. Xu , Y. Wang , C. Shi , Y. Sha , G. He , Q. Yao , K. Shao , W. Sun , J. Du , J. Fan , X. Peng , Biomaterials 2021, 278, 121167. 34624752 10.1016/j.biomaterials.2021.121167

[17] Y. Yang , S. Bernardi , H. Song , J. Zhang , M. Yu , J. C. Reid , E. Strounina , D. J. Searles , C. Yu , Chem. Mater. 2016, 28, 704.

[18] B. Liu , S. Liang , Z. Wang , Q. Sun , F. He , S. Gai , P. Yang , Z. Cheng , J. Lin , Adv. Mater. 2021, 33, 2101223. 10.1002/adma.202101223 34145652

[19] Q. Li , J. Yu , L. Lin , Y. Zhu , Z. Wei , F. Wan , X. Zhang , F. He , L. Tian , ACS Appl. Mater. Interfaces 2023, 15, 16482. 36972557 10.1021/acsami.3c00562

[20] Z. Guo , H. He , Y. Zhang , J. Rao , T. Yang , T. Li , L. Wang , M. Shi , M. Wang , S. Qiu , X. Song , H. Ke , H. Chen , Adv. Mater. 2021, 33, 2004225. 10.1002/adma.202004225 33270303

[21] Y. Yuan , C. J. Zhang , B. Liu , Angew. Chem., Int. Ed. 2015, 54, 11419. 10.1002/anie.201503640 26094980

[22] a) Y. Ma , Y. Zhang , X. Li , Y. Zhao , M. Li , W. Jiang , X. Tang , J. Dou , L. Lu , F. Wang , Y. Wang , ACS Nano 2019, 13, 11967; 31553168 10.1021/acsnano.9b06040

