# Supplementary material for: Tumor Microenvironment‐Activated Nanocomposite for Self‐Amplifying Chemodynamic/Starvation Therapy Enhanced IDO‐Blockade Tumor Immunotherapy
Source: Adv Sci (Weinh). 2023 Oct 9;10(34):2303580. doi: 10.1002/advs.202303580 (PMC10700178; doi:10.1002/advs.202303580)
Supplement: Supplementary file 1 — Supporting Information [file ADVS-10-2303580-s001.pdf]

ADVANCED SCIENCE
Open Access

Supporting Information

for *Adv. Sci.*, DOI 10.1002/advs.202303580

Tumor Microenvironment-Activated Nanocomposite for Self-Amplifying Chemodynamic/Starvation Therapy Enhanced IDO-Blockade Tumor Immunotherapy

Yulong Bian, Bin Liu, Binbin Ding, Meifang Wang, Meng Yuan, Ping'an Ma and Jun Lin**

Copyright WILEY-VCH Verlag GmbH & Co. KGaA, 69469 Weinheim, Germany, 2018.

Supporting Information

Tumor Microenvironment-Activated Nanocomposite for Self-amplifying Chemodynamic/Starvation Therapy Enhanced IDO-Blockade Tumor Immunotherapy

Yulong Bian,[a,b] Bin Liu,[a] Binbin Ding,[a] Meifang Wang,[a] Meng Yuan,[a,b] Ping'an Ma,[a,b] and Jun Lin*[a,b]*

Experimental Section

Chemicals. All the chemicals were used directly without further purification. Tetraethyl orthosilicate (TEOS), triethanolamine (TEA), sodium salicylate (NaSal), 3-aminopropyltriethoxysilane (APTES), manganese acetylacetonate [$Mn(acac)_2$], methylene blue (MB), titanic sulfate [$Ti(SO_4)_2$], glutathione (GSH), natural glucose oxidase (GOx), 3-[4,5-dimethylthia-zol-2-yl]-2,5-diphenyltetrazolium bromide (MTT), 1,2-distearoyl-sn-glycero-3-phosphoethanolamine-N-[methoxy(polyethylene glycol)] (DSPE-PEG, 3400Da) and o-phenylenediamine (OPD) were purchased from Aladdin Reagent, Ltd. (Shanghai, China). Bis(triethoxysilylpropyl) disulfide (BTES) were purchased from YuanyeBio Co., Ltd. (Shanghai, China). Oleylamine (OM) was purchased from Sigma-Aldrich (Shanghai, China). Hydrochloric acid (HCl), KH_2PO_4, methanol, ethanol, hydrogen peroxide (H_2O_2, 30%), cetyltrimethylammonium bromide (CTAB), acetic acid (HAc) and dimethyl sulfoxide (DMSO) were purchased from Sinopharm Chemical Reagent Co., Ltd (Shanghai, China). Epacadostat (IDOi) was purchased from Energy Chemical Co., Ltd (Shanghai, China). Sodium acetate (NaAc) and acridine Orange (AO) were purchased from Macklin Biochemical Co.,

Ltd (Shanghai, China). 5,5'-dithiobis (2-nitrobenzoic acid) (DTNB) were purchased from J&K Scientific Co., Ltd (Beijing, China). Recombinant Mouse IFN-γ was purchased from Solarbio Life Sciences Co., Ltd (Beijing, China).

Cell lines and animals. L929, NIH-3T3, Hela, DC 2.4, and 4T1 cells were purchased from the Cell Bank of the Chinese Academy of Sciences (Shanghai, China). The mice we used were BALB/c female mice (19-22 g) that were acquired from the Center for Experimental Animals, Jilin University (Changchun, China). All mice were handled using the protocol approved by the Institutional Animal Care and Use Committee of Jilin University (approval number: SCXK (Liao) 2020-0001).

Characterization. The morphology of the samples was determined using the S-4800 field emission scanning electron microscope (FE-SEM). The sample's crystal structure was detected by X-ray powder diffraction (Bruker) with Cu Kα radiation (λ = 0.154 nm). The transmission electron microscopy (TEM) micrographs of samples were obtained using a FEI Tecnai G2 S-Twin with a field emission gun operating at 200 kV. The UV-vis absorption spectra were measured on U-3100 spectrophotometer (Hitachi). Vertex Perkin-Elmer 580BIR spectrophotometer (Bruker) with the KBr pellet technique was employed to measure the Fourier Transform Infrared (FT-IR) spectra. The inductively coupled plasma-mass spectrometer (ICP-MS) was carried on an iCAP 6300 of Thermo scientific. X-ray photoelectron spectra (XPS) were measured on a VG ESCALAB MK II electron spectrometer using Mg Kα (1200 eV) as the excitation source. Thermogravimetry Analysis (TGA) was detected on a Netzsch Thermoanalyzer STA 409 instrument in a nitrogen environment with a heating rate of

10 °C min^{-1} from room temperature to 800 °C. Magnetic resonance imaging (MRI) was recorded on a 1.2 T MRI magnet (Shanghai Niumai Corporation Ration NM120-Analyst). The DLS and zeta potential of the samples in water were determined on a Malvern instrument Zetasizer Nano. Nitrogen adsorption-desorption isotherms were determined using a Micromeritics ASAP Tristar II 3020 system at 77 K. The electron-spin-resonance (ESR) spectra were obtained using a Bruker EMXnano spectrometer. The dissolved oxygen was measured by a dissolved oxygen meter (JPBJ-608, INESA, China).

Synthesis of dendritic mesoporous organosilica nanoparticles (DMONs). To obtain DMONs, the dendritic mesoporous organosilica nanoparticles were synthesized according to the previous reports. At first, 68 mg of TEA were added into 25 ml of water and stirred gently at 80 °C for 0.5 h in an oil bath under a magnetic stirring. Afterwards, 168 mg of NaSal and 380 mg of CTAB were added into the above mixture and keep stirring for another 1 h. After that, 2 mL TEOS and 1.6 mL BTES was added and maintained at 80 °C for 12 h. The products were collected by centrifugation and washed with deionized water and ethanol for three times to remove the residual reactants. Then, to remove the template, the collected products were extracted with HCl and methanol solution (v/v = 1:5) at 60 °C for 6 h over three times, followed by centrifugation and washing with ethanol, then drying in vacuum at 40 °C overnight to obtain DMONs.

Synthesis of Mn_3O_4@DMONs (MnD) and surface modification. To obtain MnD, 200 mg DMONs and 300 mg $Mn(acac)_2$ were added into 9.36 mL OM. Then, the

mixture was stirred to become a slurry by ultrasound. The obtained suspension was stirred for 1 h at room temperature. After that, the temperature was raised to 150 °C and kept stirring for 9 h. The resulting reaction mixture was cooled to room temperature, and the insoluble products were collected by centrifugation at 3000 rpm. The ethanol (30 mL) was added into resulting supernatant to get the other part of the product. The two parts products were mixed, followed by washing with ethanol for three times, then drying in vacuum at 40 °C overnight. For surface modification, 1 mL of APTES was added into 50 mL anhydrous ethanol containing 100 mg of MnD at 85 °C for 12 h under stirring. The synthesized products were collected by centrifugation and washed with ethanol.

Synthesis of Mn_3O_4@DMONs-IDOi (MnDI). First, 30 mg of MnD and 5 mg IDOi were dispersed in 5 mL of dimethyl sulfoxide. Then, the mixture was kept stirring for 12 h at room temperature. The resulting products were collected by centrifugation and washed with water for three times. The synthesis procedure of DMONs-IDOi (DI) was same to MnDI.

Synthesis of Mn_3O_4@DMONs-IDOi-GOx (MnDIG). First, 5 mg of MnDI and 5 mg GOx were dispersed in 5 mL of water. Then, the mixture was kept stirring for 12 h at room temperature. The resulting products were collected by centrifugation and washed with water for three times. The synthesis procedure of DMONs-GOx (DG), Mn_3O_4@DMONs-GOx (MnDG) was same to MnDIG.

Synthesis of DSPE-PEG modified MnDIG (MnDIG@PEG)

50 mg of MnDIG NPs were evenly dispersed in 20 mL of water under ultrasound. Afterwards, 50 mg of DSPE-PEG was added to the above solution and kept stirring for 12 h at room temperature. The product was collected by centrifugation and freeze-dried.

The loading efficiencies of IDOi and GOx. To evaluate the loading efficiencies of IDOi, we measured the concentrations of IDOi in the supernatant by the high performance liquid chromatography (HPLC). Similarly, the concentration of GOx was determined by a UV-vis spectrophotometer (at 450 nm). The loading efficiencies were calculated using the following equation:

$$\textbf{Load capacity}=\frac{m_{original}-m_{supernatant}}{M_{nanocarrier}+m_{original}-m_{supernatant}}$$

$m_{original}$ was the amount of original IDOi or GOx (mg); $m_{supernatant}$ was the amount of IDOi or GOx in the supernatant (mg); $M_{nanocarrier}$ was the amount of MnD or MnDI NPs (mg). So, the loading efficiencies of IDOi and GOx were evaluated to be 2.7% and 20.8%, respectively.

Stability evaluation. To evaluate the colloidal stability, the particle size of MnDIG@PEG in PBS, FBS, RPMI-1640 medium, and acid H_2O_2 solution (pH 5.5) were measured from day 1 to day 7 by dynamic light scattering (DLS).

***In vitro* biodegradation behavior test.** To study the performance of MnDIG@PEG in different environments, 1 mg mL^{-1} of MnDIG@PEG with GSH (10 mM) were dispersed in phosphate buffer saline (PBS, pH 7.4) or sodium acetate-acetate buffer (pH 5.5) at 37 °C for 6, 12, 24, 72, and 120 h. Then, the TEM assays were performed for the degradation test. Moreover, 1 mg mL^{-1} of MnDIG@PEG with different

concentration of GSH (0.1, 0.2, 0.4, 0.8, 1.6, 2.4, 3.2, and 4 mM) were dispersed in sodium acetate-acetate buffer (pH 5.5) at 37 °C for 0.5 h. The absorbance of solution was measured by a UV-vis spectrophotometer.

pH/GSH-triggered release behavior test. 1 mg mL^{-1} of MnDIG@PEG with or without 10 mM GSH were incubated with different pH solutions (pH 7.4, 6.5, and 5.5) at 37 °C. After mixing them for 1, 3, 6, 12, 24, and 48 h, followed by centrifugation and collect the supernatant. The released Mn^{2+}, IDOi, and GOx were determined by ICP-MS, HPLC, or UV-vis absorption spectra.

Peroxidase-mimic activity measurement of MnDIG@PEG. Peroxidase-mimic activity assays of MnDIG@PEG were performed using OPD or MB as substrate. In brief, the MnDIG@PEG (1 mg mL^{-1}) with 1 mM GSH were dispersed in sodium acetate-acetate buffer (pH 5.5) and incubated for 1 h at 37 °C. After centrifugation and collect the supernatant, H_2O_2, $NaHCO_3$/5% CO_2 buffer (25 mM), MB or OPD were then added for another 30 min of incubation ($[H_2O_2]$ = 0, 1, 2, 4, 8, and 10 mM). Subsequently, the absorbance of the color reactions was recorded by absorbance change of 665 nm or 417 nm using a UV-vis spectrophotometer.

For the peroxidase-mimic activity of MnDIG@PEG at different concentrations of GSH ([GSH] = 0, 1, 2, 4, 8, and 10 mM) or glucose ([glucose] = 0, 1, 2, 5, and 10 mM) was detected the absorbance change using MB or OPD as the substrate in the presence of 1 mM H_2O_2, $NaHCO_3$/5% CO_2 buffer (25 mM) at different concentrations of GSH or glucose.

ESR measurement of •OH. For detection of •OH generation, 5,5-dimethyl-1-pyrroline-N-oxide (DMPO) was used as a trapping probe to capture •OH by ESR spectrum. Mn^{2+} (100 μM), H_2O_2 (2 mM), $NaHCO_3$/5% CO_2 buffer (25 mM), glucose (10 mM) and DMPO were used to measure •OH after 30 min reaction.

GSH depletion measurement. MnDIG@PEG (1 mg mL^{-1}) with different concentration of GSH ([GSH] = 0, 1, 2, 4, 8, and 10 mM) were dispersed in sodium acetate-acetate buffer (pH 5.5) and then incubated for 1 h at 37 °C, followed by centrifugation and collecting the supernatant. Then, DTNB was added to detect the -SH group in GSH. The absorbance changes at 412 nm were measured using UV-vis spectroscopy after 5 min of incubation.

H_2O_2 consumption measurement. For detection of H_2O_2 consumption, $Ti(SO_4)_2$ was used as an indicator of H_2O_2. MnDG (1 mg mL^{-1}) with 1 mM H_2O_2 were dispersed in sodium acetate-acetate buffer (pH 5.5) and then incubated for different time (30, 60, 90, 120, 150, and 180 min), followed by centrifugation and collecting the supernatant. After that, $Ti(SO_4)_2$ (500 μg mL^{-1}) was added to detect H_2O_2. The absorbance changes at 410 nm were measured using UV-vis spectroscopy after 1 min of incubation.

Detection of O_2 production or consumption ability. For detection the concentration of O_2, we used a dissolved O_2 meter. First, 10 ml of different samples solution ([Mn^{2+}] = 100 μM) was added to a 50 mL centrifuge tube with constant stirring. Then, 100 μL of H_2O_2 (100 mM) or glucose (1 M) was injected into the solution, followed by recording the concentration of O_2.

Detection of glucose consumption ability.

For detection the concentration of glucose, we used a glucose test kit. First, 1 ml of different samples solution ([Mn^{2+}] = 100 μM) or GOx was added to a 4 mL centrifuge tube with glucose (10 mM) at pH 5.5 sodium acetate-acetate buffer and then incubated for different time (30, 60, 120, 240, and 360 min), followed by centrifugation and collecting the supernatant. After that, the concentration of glucose was detected as the specification of the kit.

Detection of pH level catalyzed by GOx. In brief, 10 ml of different samples solution ([Mn^{2+}] = 100 μM) was added to a 50 mL centrifuge tube with constant stirring. Then, 100 μL of glucose (1 M) was injected into the solution, followed by recording the pH level of solution.

***In vitro* Cytotoxicity Evaluation.** L929 and DC 2.4 cells were chosen to evaluate the biocompatibility of MnDIG@PEG. In brief, the cells were seeded in 96-well plates at a density of 6000 cells per well. Then, different concentrations of MnDIG@PEG ([Mn^{2+}] = 0, 6.25, 12.5, 25, 50, and 100 μM) were added and incubated for 24 h. At the end of incubation, we use the standard 3-(4,5-dimethylthiazol-2-yl)-2,5-diphenyltetrazolium bromide (MTT) assay to measure relative cell viabilities.

In vitro cytotoxicity of different samples was assayed against 4T1 cells by MTT assay. The cells were seeded in 96-well plates at a density of 6000 cells per well. Then, the wells were divided into six groups: (1) control, (2) DI, (3) DG, (4) MnD, (5) MnDG, (6) MnDIG@PEG ([Mn^{2+}] = 0, 6.25, 12.5, 25, 50, and 100 μM). Then, we use the standard MTT process to assess cell viability at different concentrations of samples after incubating for 24 h. The cooperative index (CI) was calculated according to the

equation of CI = $DA/IC_{50(A)} + DB/IC_{50(B)}$, in which $IC_{50(A)}$ and $IC_{50(B)}$ are the respective IC_{50} values of drugs A and B in the presence of drug A or B alone, as well as DA and DB are drug concentrations of drugs A and B to reach IC_{50} in the presence of both drugs, respectively. CI < 0.8 represents a highly significant cooperative effect, and CI < 1.0 is considered as a significant cooperative effect.

***In vitro* cellular uptake efficacy of MnDIG@PEG.** Cellular uptake efficacy was demonstrated by ICP-MS and intracellular fluorescence. 4T1 cells were seeded into 6-well plates at a density of 8 × 10^4 cells per well incubated with MnDIG@PEG ($[Mn^{2+}]$ = 25 μM) for 0.5, 1, 3, and 6 h before the cells were imaged by inverted fluorescence microscope (Nikon Ti–S). Moreover, 4T1 cells seeded into 6 well plates at a density of 8 × 10^4 cells per well incubated with MnDIG@PEG ($[Mn^{2+}]$ = 25 μM) for 0.5, 1, 3, 6, 8 and 12 h, respectively. After washing with PBS, the cells were collected and observed by bio-TEM. In addition, 4T1 cells seeded into 6 well plates at a density of 8 × 10^4 cells per well incubated with MnDIG@PEG ($[Mn^{2+}]$ = 25 μM) for 0.5, 1, 3, and 6 h, respectively. After washing with PBS, the cells were lysed using cell lysis buffer. The concentration of Mn was measured by ICP-MS.

***In vitro* GSH and H_2O_2 levels Measurement.** 4T1 cells were seeded into culture plates at a density of 3 × 10^5 cells per well and treated with different nanomaterials (DI, DG, MnD, MnDG, and MnDIG@PEG, $[Mn^{2+}]$ = 25 μM). The amounts of H_2O_2 and GSH were detected employing H_2O_2 and GSH assay kits following its protocol after another 6 h of incubation.

***In vitro* ROS generation Measurement.** To evaluate the ROS levels of cells, the 4T1 cells were planted into 6-well plates overnight and they were co-incubated with DI, DG, MnD, MnDG, MnDIG@PEG and MnDIG@PEG + glucose ([Mn^{2+}] = 25 μM) for another 6 h. After incubation, the DCFH-DA was added to each well and incubated for 30 min at 37 ^{o}C. Finally, the cells were measured by the intracellular fluorescence intensity of DCF (Ex: 485 nm, Em: 545 nm) to test the production of ROS.

***In vitro* LPO accumulation Measurement.** To evaluate the LPO levels of cells, 4T1 cells were seeded into culture plates at a density of 1 × 10^6 cells per well and treated with different concentration MnDIG@PEG for 24h. The cells were collected and treated with cell lysis buffer in the presence of protease and phosphatase inhibitor cocktail. The LPO content was studied by utilizing lipid peroxidation assay kit.

***In vitro* lysosomal membrane integrity Measurement.** Lysosomal membrane integrity of 4T1 cells after incubation with different nanomaterials was evaluated by the acridine orange (AO) staining method. The 4T1 cells were planted into 6-well plates overnight. After that, they were co-incubated with DI, DG, MnD, MnDG, and MnDIG@PEG ([Mn^{2+}] = 25 μM) for another 6 h. After incubation, the AO was added to each well and incubated for 5 min at 37 ^{o}C. Finally, the cells were measured by inverted fluorescence microscope.

***In vitro* mitochondrial integrity Measurement.** The 4T1 cells were planted into 6-well plates overnight. After that, they were co-incubated with DI, DG, MnD, MnDG, and MnDIG@PEG ([Mn^{2+}] = 25 μM) for another 6 h. Finally, the mitochondrial

integrity was detected employing mitochondrial integrity assay kits following its protocol by inverted fluorescence microscope.

***In vitro* O_2 level Measurement.** To evaluate the O_2 levels of cells, the 4T1 cells were planted into 6-well plates overnight under hypoxia. After incubation, the RDPP (10 μM) was added to each well and incubated overnight. Then, they were co-incubated with DI, DG, MnD, MnDG, and MnDIG@PEG ([Mn^{2+}] = 25 μM) for another 6 h under the same condition. Finally, the cells were measured by inverted fluorescence microscope.

***In vitro* glucose level Measurement.** To evaluate the glucose levels of cells, the 4T1 cells were planted into 6-well plates overnight. After incubation, they were co-incubated with DI, DG, MnD, MnDG, and MnDIG@PEG ([Mn^{2+}] = 25 μM) for another 6 h under the same condition. Finally, the amounts of glucose were detected employing glucose assay kits following its protocol.

***In vitro* calreticulin (CRT) expression Measurement.** To evaluate the CRT expression levels of cells, the 4T1 cells were planted into 6-well plates overnight. After incubation, they were co-incubated with DI, DG, MnD, MnDG, and MnDIG@PEG ([Mn^{2+}] = 25 μM) for another 12 h under the same condition. After that, the cells were washed repeatedly with PBS and then incubated with Alexa Fluor 647-CRT antibody (RabMAb, ab196159) for 30 min and detected by flow cytometer.

***In vitro* High-mobility group box 1 (HMGB1) and adenosine triphosphate (ATP) Measurement.** The extracellular released HMGB1 and ATP were examined using the HMGB1 enzyme-linked immunosorbent assay (ELISA) Kit (CUSABIO, CSB-

E08225m) and ATP Assay Kit (Beyotime, A22066), respectively. Briefly, 4T1 cells were seeded into 6-well culture plates at a density of 3 × 10^5 cells per well overnight. After incubation, they were treated with DI, DG, MnD, MnDG, and MnDIG@PEG ([Mn^{2+}] = 25 μM) for another 12 h under the same condition. After that, the cell supernatant was collected *via* a centrifugation at 2000 rpm for 5 min. The release of HMGB1 or ATP in the cell supernatant was detected by the HMGB1 ELISA Kit or ATP Assay Kit according to the manufacturer's protocols.

***In vitro* IDO inhibitory effect Measurement.** To measure the IDO inhibitory effect of MnDIG@PEG, 4T1 cells were seeded in the 96-well plate at a density of 5000 cells per well. After 24 h incubation, IFN-γ was added to each well to a final concentration of 100 ng ml^{-1}. Meanwhile, the cells were treated with DI, DG, MnD, MnDG, and MnDIG@PEG. After 12 h incubation, 150 μL of the supernatant from each well was pipetted into a new 96-well plate and incubated with trichloroacetic acid (75 μL, 30%) at 50 °C for 30 min to hydrolyse N-formylkynurenine to kynurenine. Finally, 100 μL supernatant was placed in another 96-well plate where 100 μL of Ehrlich reagent (2% p-dimethylamino-benzaldehyde w/v in glacial acetic acid) was added for 15 min at room temperature. The absorbance of the reaction product was measured at 490 nm using a microplate reader.

Cell apoptosis Analysis. For apoptosis analysis by flow cytometry, in brief, the cells seeded in a 6-well plate at a density of 2 × 10^5 cells per well. The cells were treated with the same protocols as the above mentioned MTT assay, and all floating and adherent cells were collected after 24 h of incubation. After using the Annexin V

FITC/PI stain the live and dead cells for 15 min, the flow cytometer was used to determined cell apoptosis.

***In vitro* and *in vivo* MRI Evaluation of MnDIG@PEG.** Different concentrations of MnDIG@PEG ([Mn^{2+}] = 0, 6.25, 12.5, 25, 50, and 100 μM) were dispersed in different pH solution PBS (pH 7.4) or sodium acetate-acetate buffer (pH 5.5) and incubated for 24 h. The supernatant was collected through centrifugation and the MRI signals were measured by MRI scanner. *In vivo* MRI experiments were acquired by intravenous injection of MnDIG@PEG (20 mg kg^{-1}) and the corresponding MRI was measured.

***In vivo* pharmacokinetic evaluation.** Female BALB/c mice were subcutaneously injected with 4T1 cells. All mice were intravenously injected with MnDIG@PEG (20 mg kg^{-1}) for 0.5, 1, 2, 4, 8, 12, and 24 h post injection, then the mice were sacrificed. Major organs (heart, liver, spleen, lung, and kidneys) and tumors were dissected, weighed, and dissolved with aqua regia solution. The bio-distribution in different organs and tumors were calculated as the Mn^{2+} percentage of injected dose per gram of tissues. Furthermore, the *in vivo* fluorescence images were captured at 1, 3, 6, 12, and 24 h post-injection using a Maestro In-Vivo Imaging System. In addition, female BALB/c mice were intravenously injected with MnDIG@PEG. After 0.5, 1, 2, 4, 8, 12, and 24 h, blood or faeces were obtained and diluted to 1 mL with PBS or aqua regia solution. The concentrations of Mn^{2+} were measured by ICP-MS. The blood circulating half-life of MnDIG@PEG ($\tau_{1/2}$) was determined using a compartment pharmacokinetic model.

***In vivo* anti-tumor effect Evaluation.** Healthy female BALB/c mice (19-22 g) were purchased from Changchun Institute of Biological Products and all mice were handled using the protocol approved by the Institutional Animal Care and Use Committee of Jilin University. Before starting the tumor-suppressive experiments, the *in vivo* biocompatibility evaluation was first investigated. The healthy female BALB/c mice were injected with MnDIG@PEG (0 and 20 mg kg^{-1}). The major organs in the control and treatment groups were fixed with paraformaldehyde to stain by H&E after 0, 7, 14, and 30 days of treatment, respectively. After being recorded for 30 days, the mice were euthanized. The blood of the control and treatment groups were collected to carry out the serum biochemistry assays.

The tumor-bearing mice were randomized into seven groups (n = 5, each group) and were treated by intravenous injection with i: PBS, ii: DI, iii: DG, iv: MnD, v: MnD@PEG vi: MnDG, vii: MnDIG@PEG. The tumor sizes were measured every 2 days and body weights were recorded at the same time. Then the mice were euthanized at 14 days' post-injection and the tumors in each group were removed and weighed. Flow cytometry was used to analyze $CD8^+$ T cells, $CD4^+$ T cells, DCs, and Treg cells in splenocytes and tumors. Lungs were also collected for studying lung metastasis when mice were sacrificed on days 28.

Histological examination. Two weeks of therapy later, the major organs (heart, liver, spleen, lung, and kidney) and tumors were collected from groups i-vii. Next, the H&E assay was used to confirm whether MnDIG@PEG could cause injury to the organs or not. At the same time, H&E, TUNEL, Ki67, ROS, CRT, HMGB1 and IDOi assays of

tumors collected from groups i-vi were used to assess *in vivo* anticancer effect of MnDIG@PEG.

Statistical analysis. Quantitative data were indicated as mean ± S.D. Means were compared using the student's t-test. The sample size was 3 or 5. And the SPSS Statistics was used for statistical analysis. Statistical significance was assumed at a value of $*p < 0.05$, $**p < 0.01$, $***p < 0.001$.

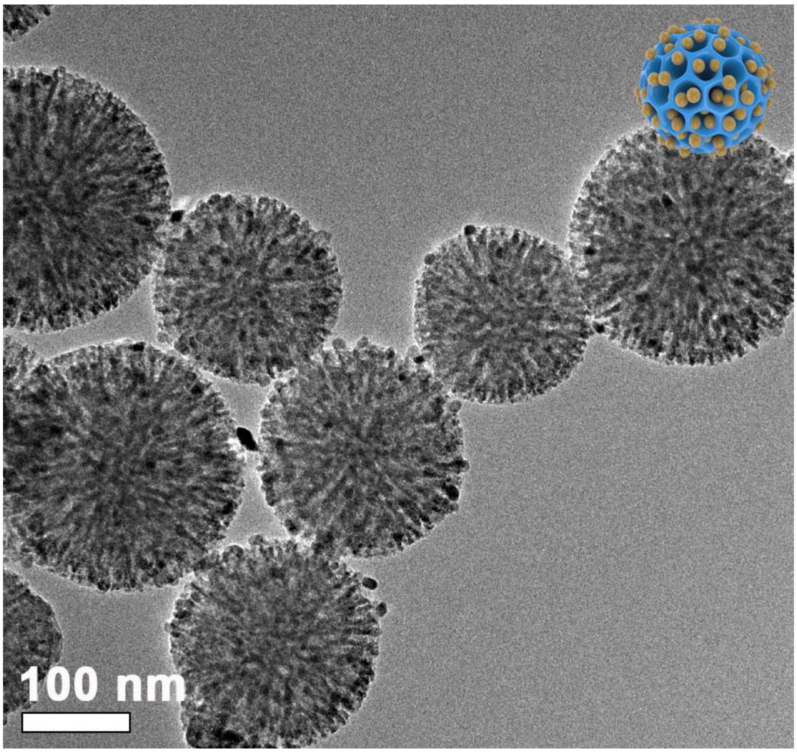

Figure S1. TEM image of as-prepared MnD.

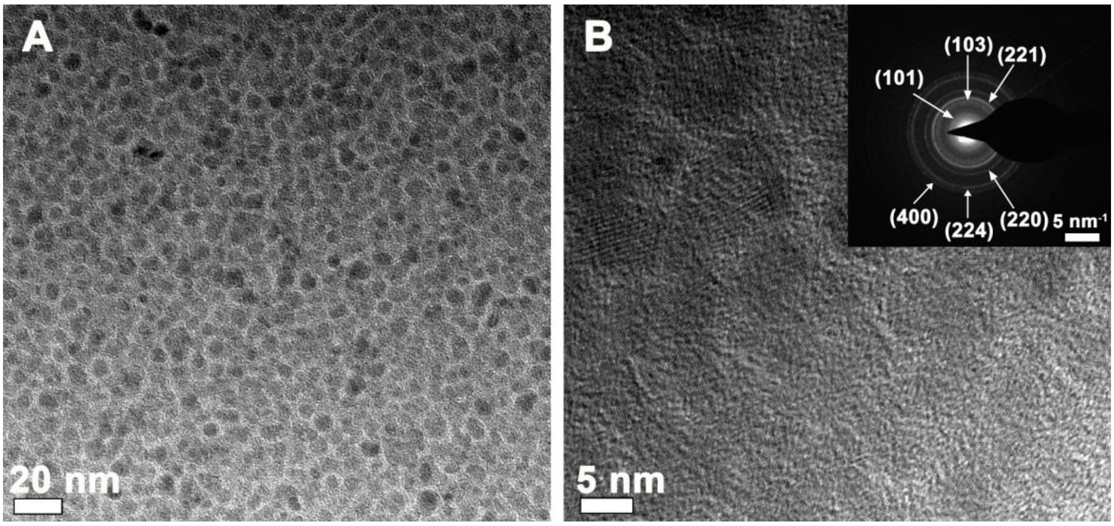

Figure S2. (A) TEM image of as-prepared Mn_3O_4 NPs. (B) High resolution TEM image of as-prepared Mn_3O_4 NPs (inset: the selected-area electron-diffraction (SAED) pattern of as-prepared Mn_3O_4 NPs).

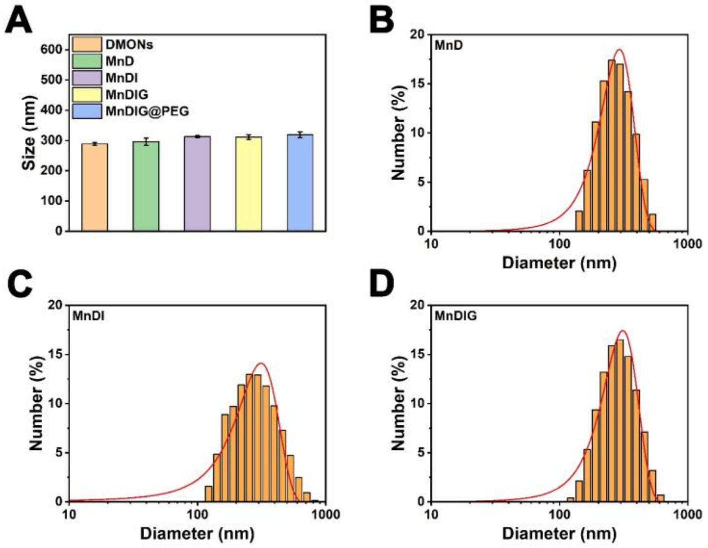

Figure S3. The size distribution of (A) various formulations, (B) MnD (C) MnDI, and (D) MnDIG (n = 3, mean ± SD).

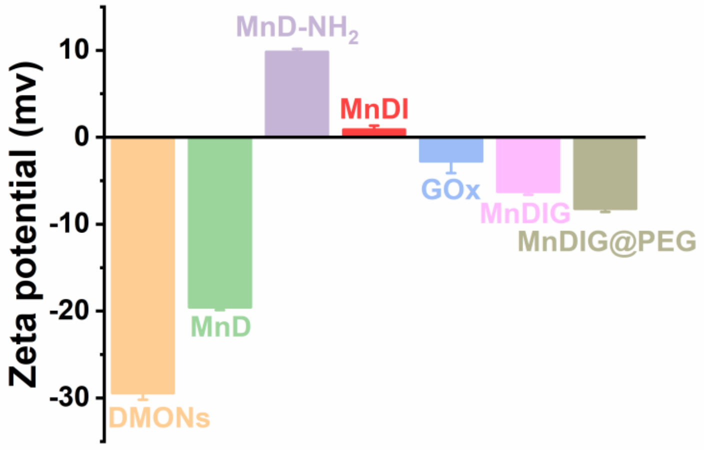

Figure S4. The zeta potential of various formulations (n = 3, mean ± SD).

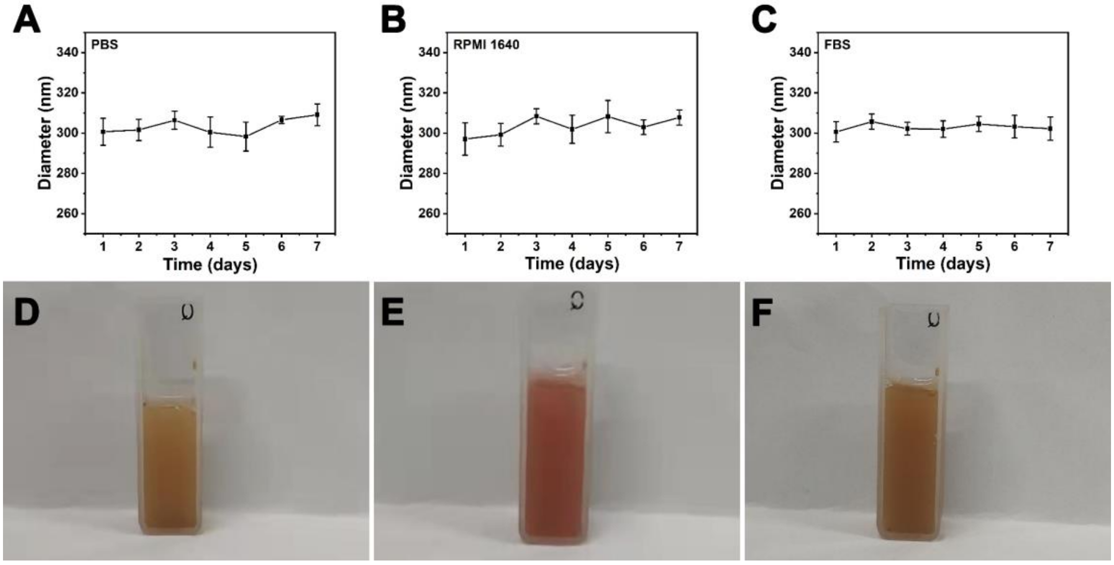

Figure S5. The DLS diameter of MnDIG@PEG in (A) PBS, (B) RPMI-1640, and (C) Fetal Bovine Serum from day 1 to day 7, respectively. The corresponding digital photograph images of MnDIG@PEG in (D) PBS, (E) RPMI-1640, and (F) Fetal Bovine Serum, respectively.

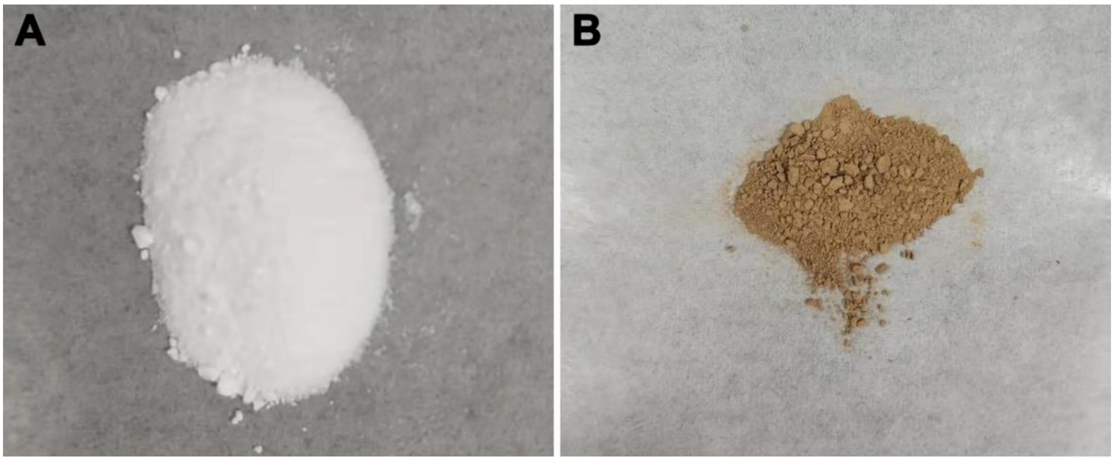

Figure S6. The digital photographs of as-prepared (A) DMONs, (B) MnDIG@PEG.

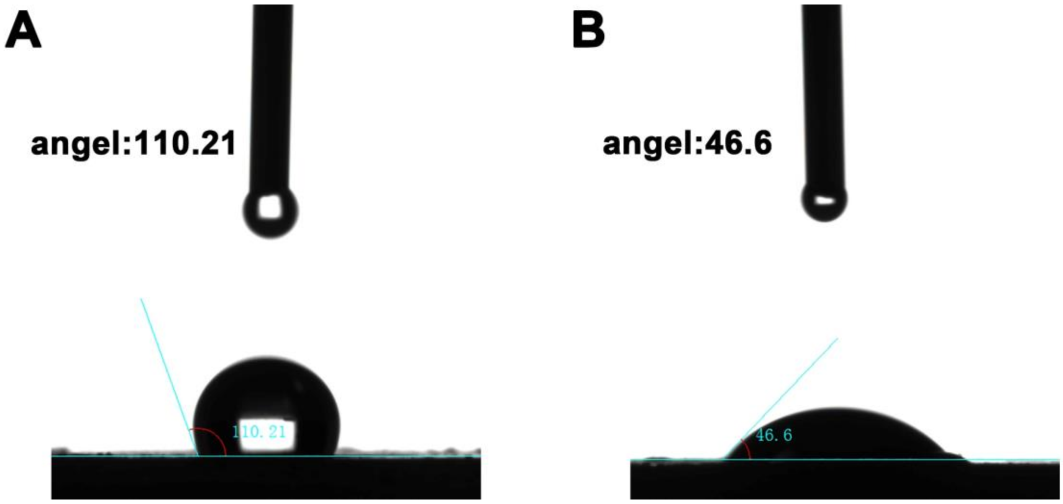

Figure S7. The contact angle of (A) MnDIG, and (B) MnDIG@PEG.

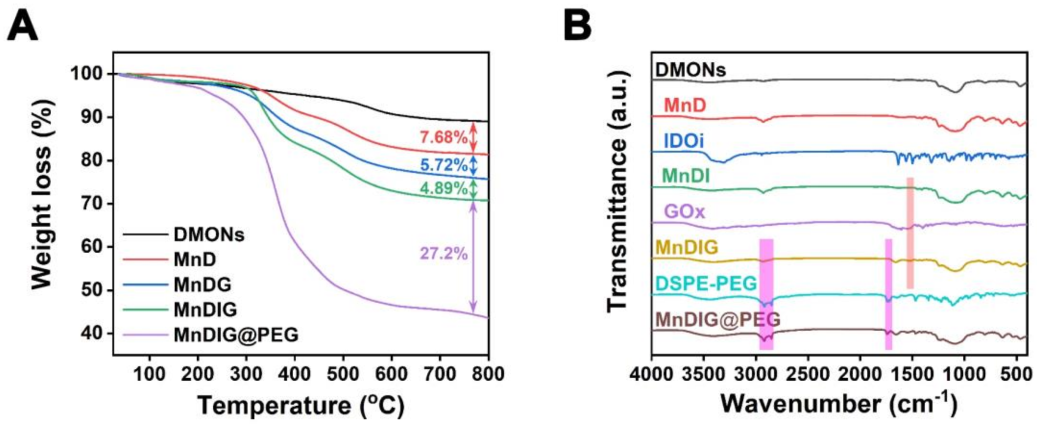

Figure S8. (A) TGA curves, and (B) FT-IR spectra of various formulations.

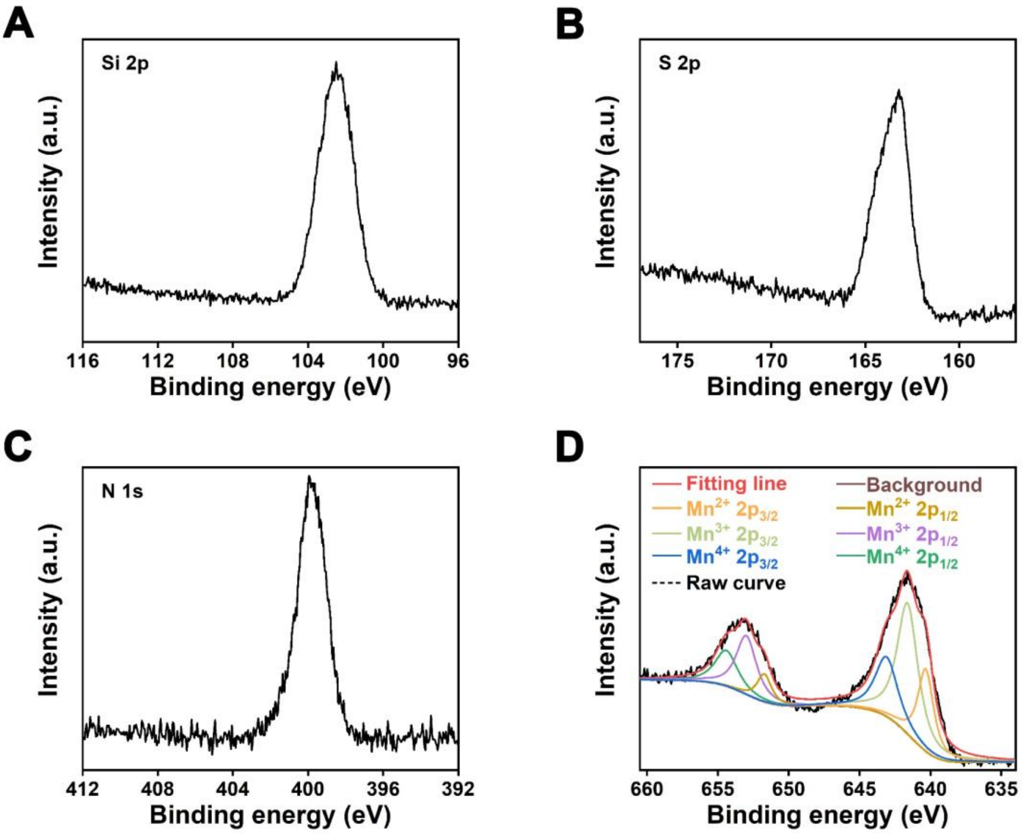

Figure S9. The XPS high-resolution scans of (A) Si 2p and (B) S 2p peaks in DMONs; (C) N 1s and (D) Mn 2p peaks in MnDIG.

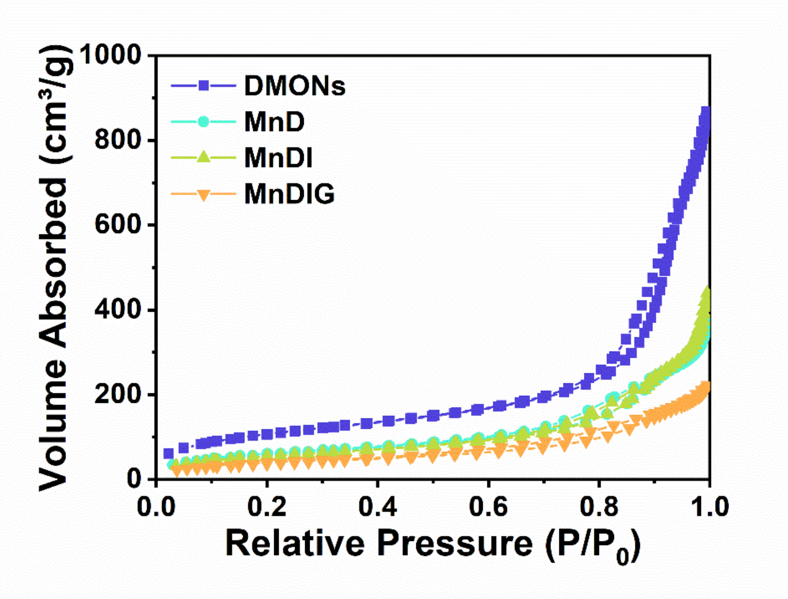

Figure S10. N_2 adsorption-desorption isotherm of various formulations.

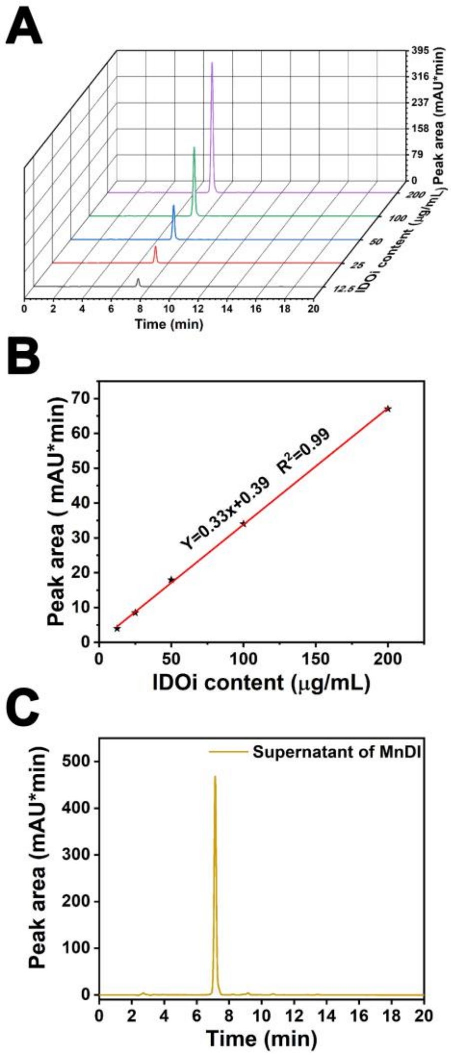

Figure S11. (A) High performance liquid chromatography of free IDOi solution at different concentrations. (B) The standard curve of IDOi. (C) High performance liquid chromatography of the supernatant obtained from centrifugation after stirring between MnDI solution.

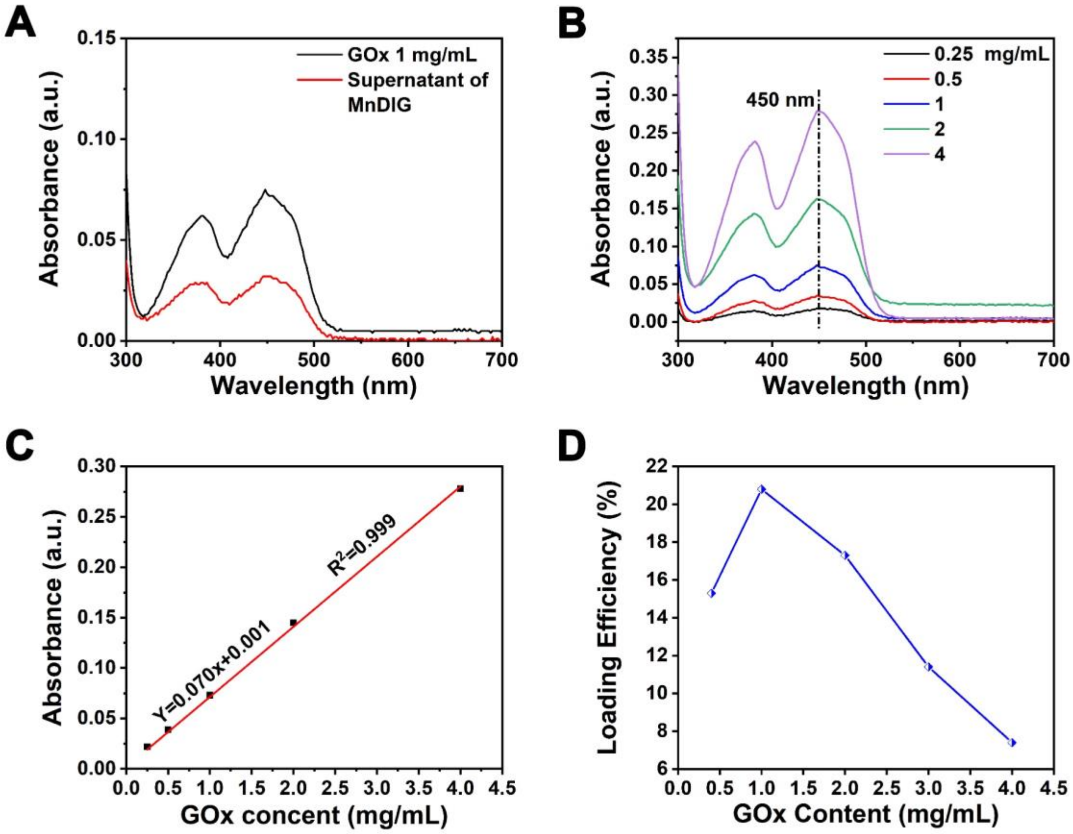

Figure S12. (A) UV-vis spectra of free GOx and the supernatant obtained from centrifugation after stirring between MnDIG aqueous solution. (B) UV-vis spectra of free GOx solution at different concentrations. (C) The standard curve of GOx. (D) The loading efficiency of GOx after stirring between MnDIG aqueous solution under different GOx concentration.

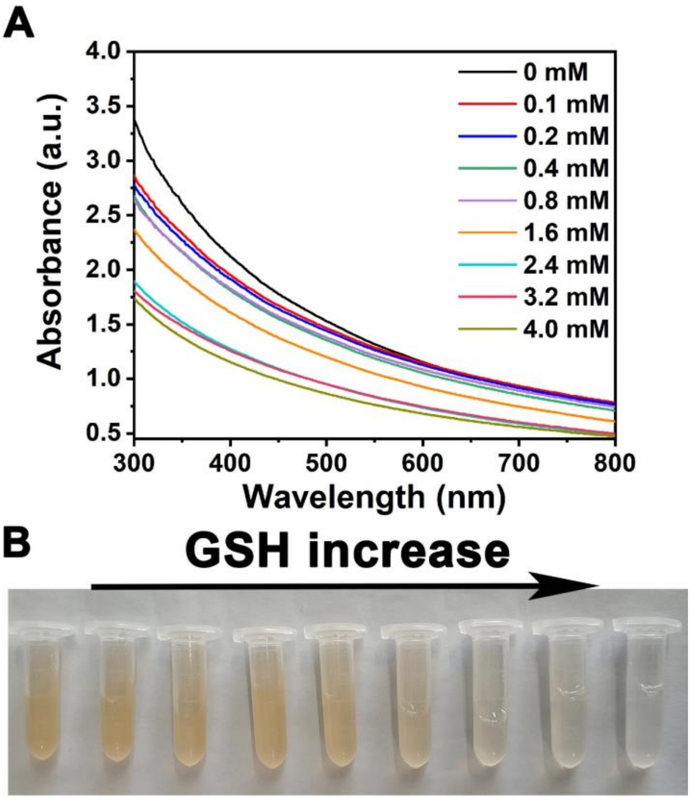

Figure S13. (A) UV-vis spectra of MnDIG@PEG solution reacted with different concentrations of GSH. (B) The corresponding digital photograph of MnDIG@PEG solution reacted with different concentrations of GSH.

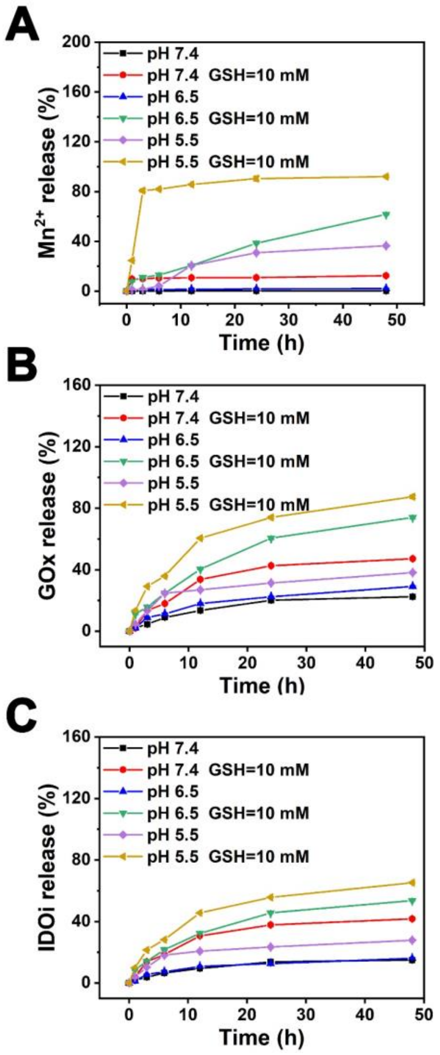

Figure S14. (A) Mn^{2+}, (B) GOx, and (C) IDOi release profiles from MnDIG@PEG nanoparticles treated by GSH in different conditions (n = 3, mean ± SD).

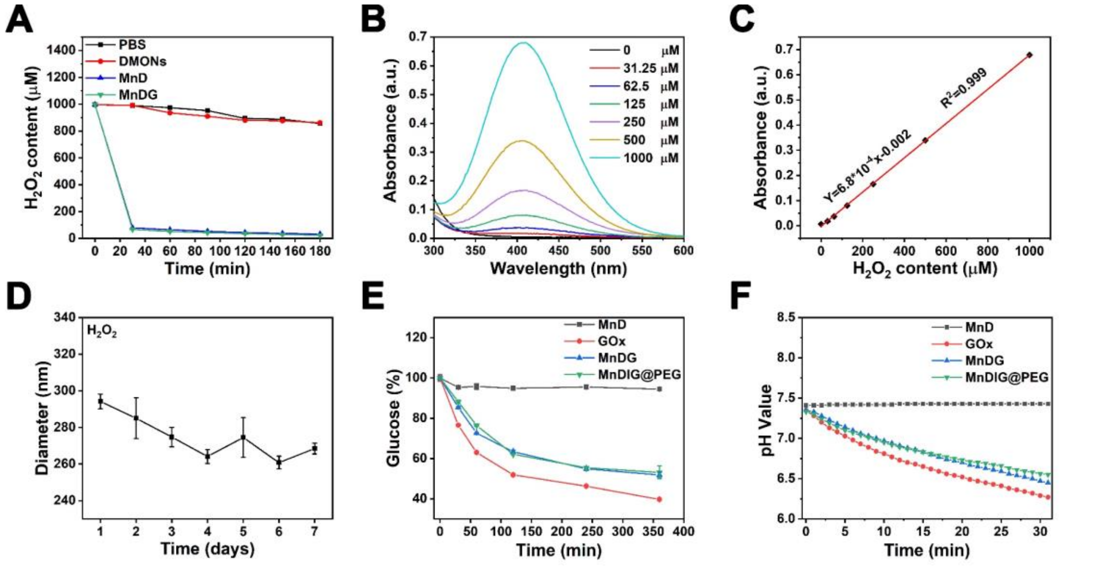

Figure S15. (A) UV-vis spectra of $Ti(SO_4)_2$ solution reacted with different concentrations of H_2O_2 solution. (B) H_2O_2 consumption profiles of different samples. (C) The standard curve of H_2O_2. (D) The DLS diameter of MnDIG@PEG in H_2O_2 solution day 1 to day 7. (E) Glucose consumption profiles of different samples. (F) pH value change profiles of different samples in the presence of glucose.

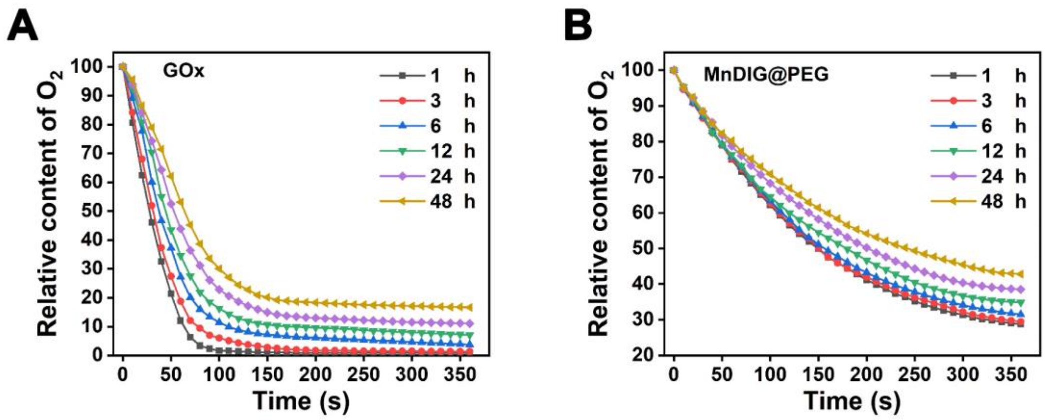

Figure S16. O_2 consumption profiles of (A) GOx, and (B) MnDIG@PEG in the presence of glucose during different incubation time (pH = 5.5, GSH = 10 mM).

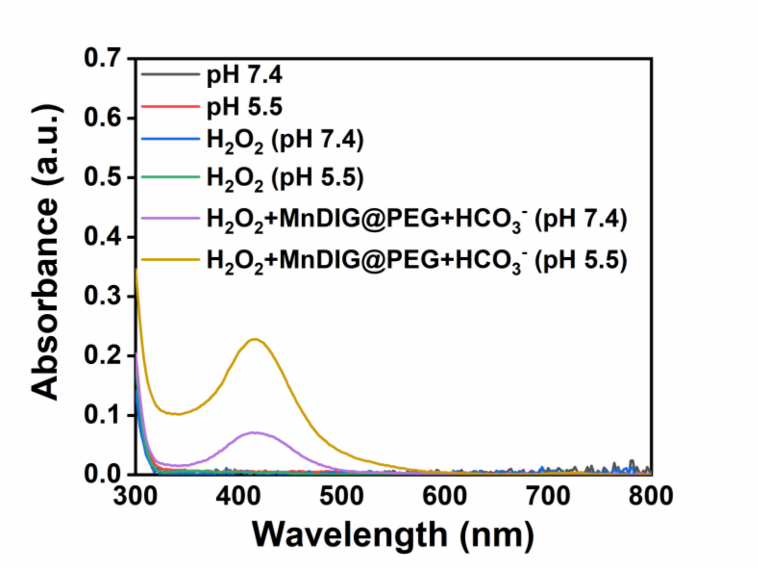

Figure S17. UV-vis absorption spectra of the OPD incubated with H_2O_2 plus MnDIG@PEG treated with pH 7.4 and 5.5.

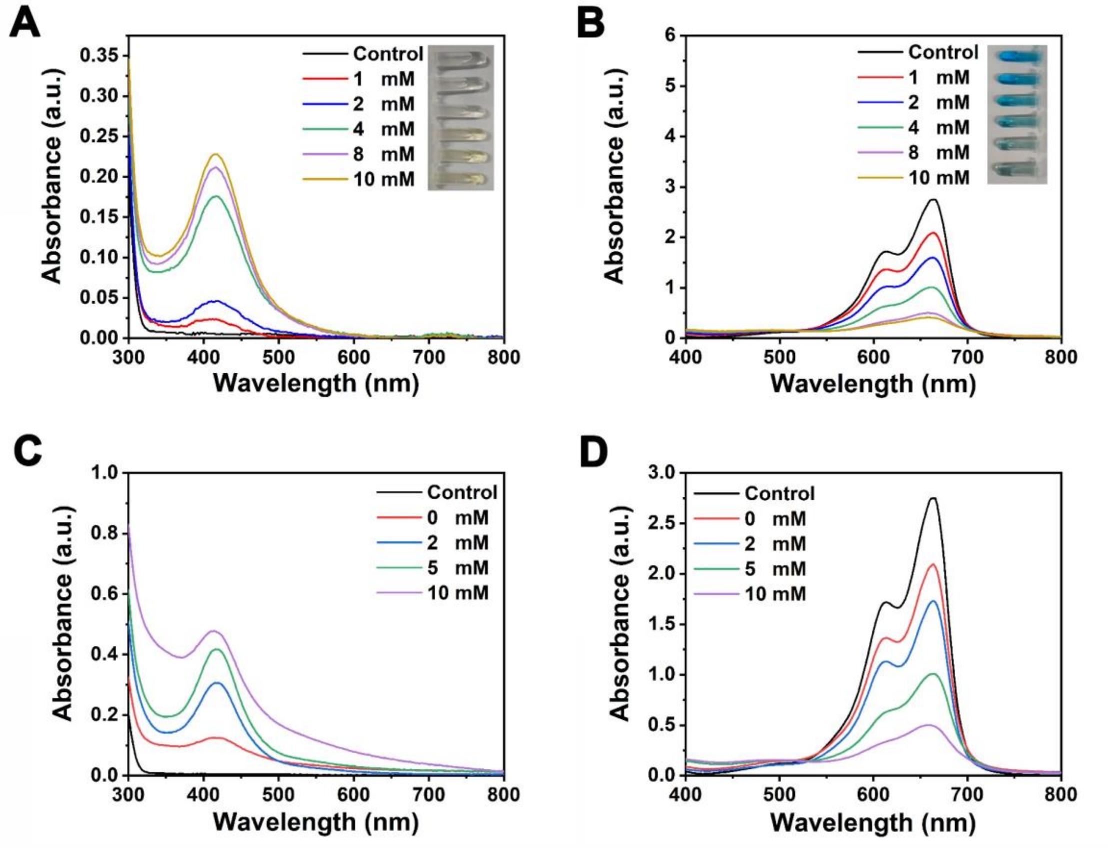

Figure S18. (A) UV-vis spectra of the OPD (oxOPD) incubated with different concentrations of H_2O_2 plus GSH-treated MnDIG@PEG (inset: the corresponding digital photograph). (B) UV-vis spectra of the MB incubated with different concentrations of H_2O_2 plus GSH-treated MnDIG@PEG (inset: the corresponding digital photograph). UV-vis absorption spectra of the (C) OPD and (D) MB incubated with MnDIG@PEG under different concentration of glucose.

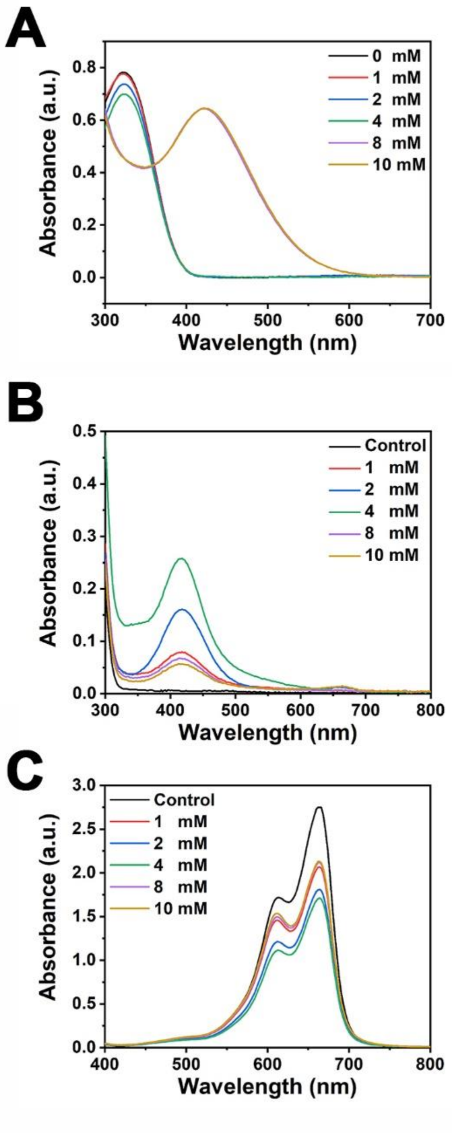

Figure S19. (A) UV-vis absorption spectra of degradation of DTNB by unreacted GSH after treatments with various concentrations of GSH plus acid-incubated MnDIG@PEG. UV-vis absorption spectra of the (B) OPD and (C) MB incubated with MnDIG@PEG under different concentration of GSH.

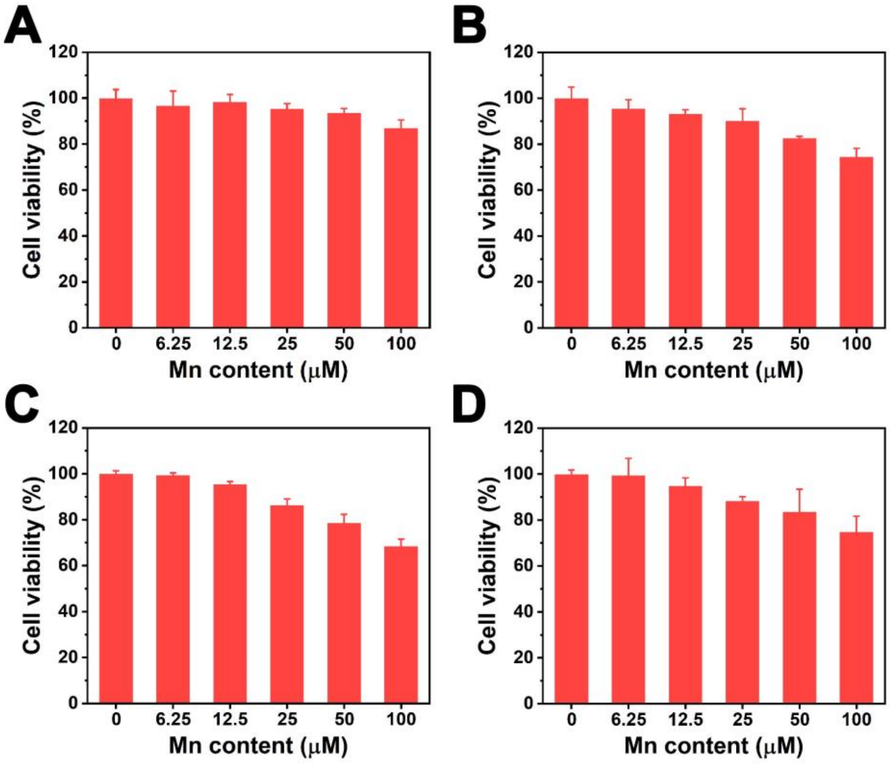

Figure S20. Cell survival rates of L929 cells incubated with (A) MnD, and (B) MnDIG@PEG for 24 h at 37 °C. Cell survival rates of (C) DC 2.4 cells, and (D) NIH-3T3 cells incubated with MnDIG@PEG for 24 h at 37 °C.

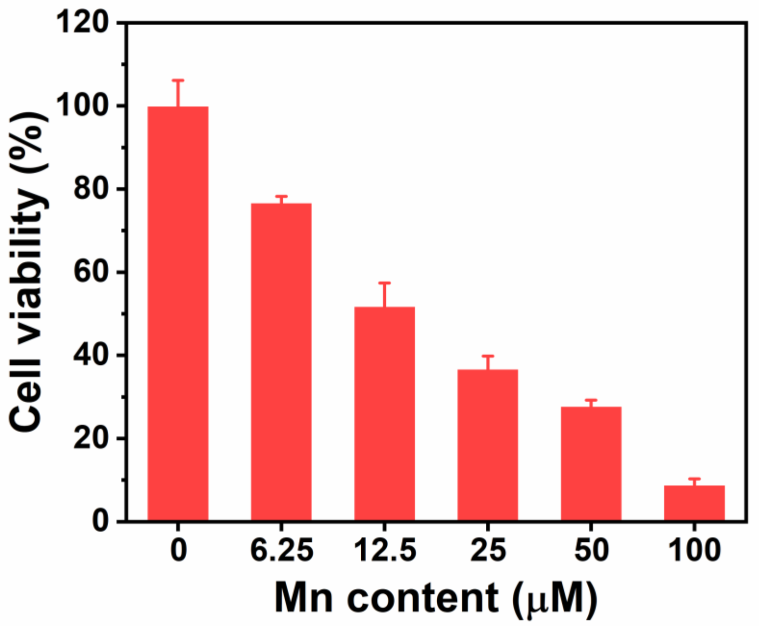

Figure S21. Cell survival rates of Hela cells incubated with MnDIG@PEG for 24 h at 37 °C.

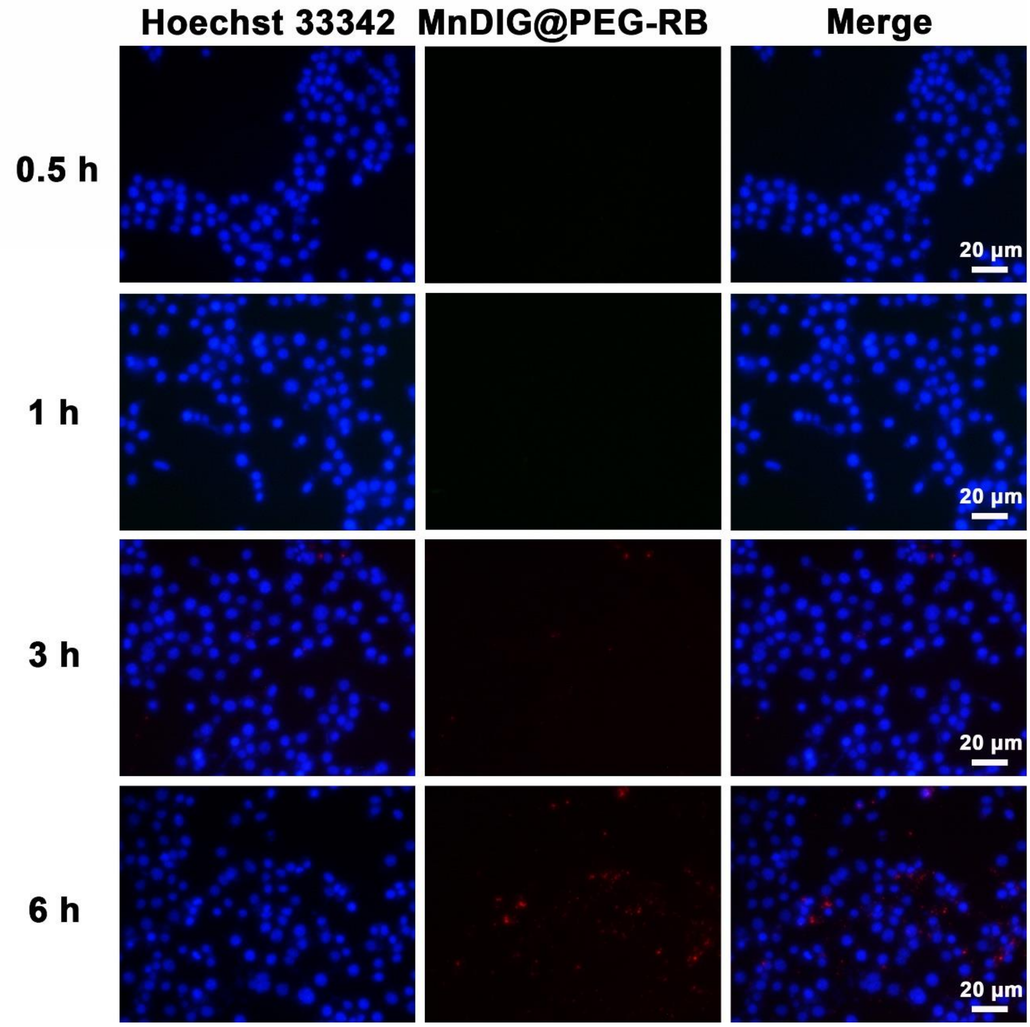

Figure S22. Cellular endocytosis of MnDIG@PEG-RB in 4T1 cells after incubating for 0.5, 1, 3, and 6 h at 37 °C, respectively. Scale bars: 20 μm.

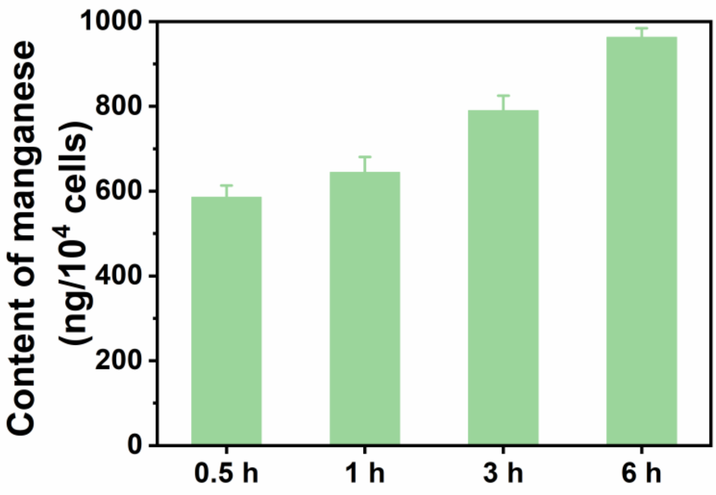

Figure S23. The mass of manganese internalized in 4T1 cells after incubating with MnDIG@PEG-RB in 4T1 cells for 0.5, 1, 3, and 6 h at 37 °C, respectively.

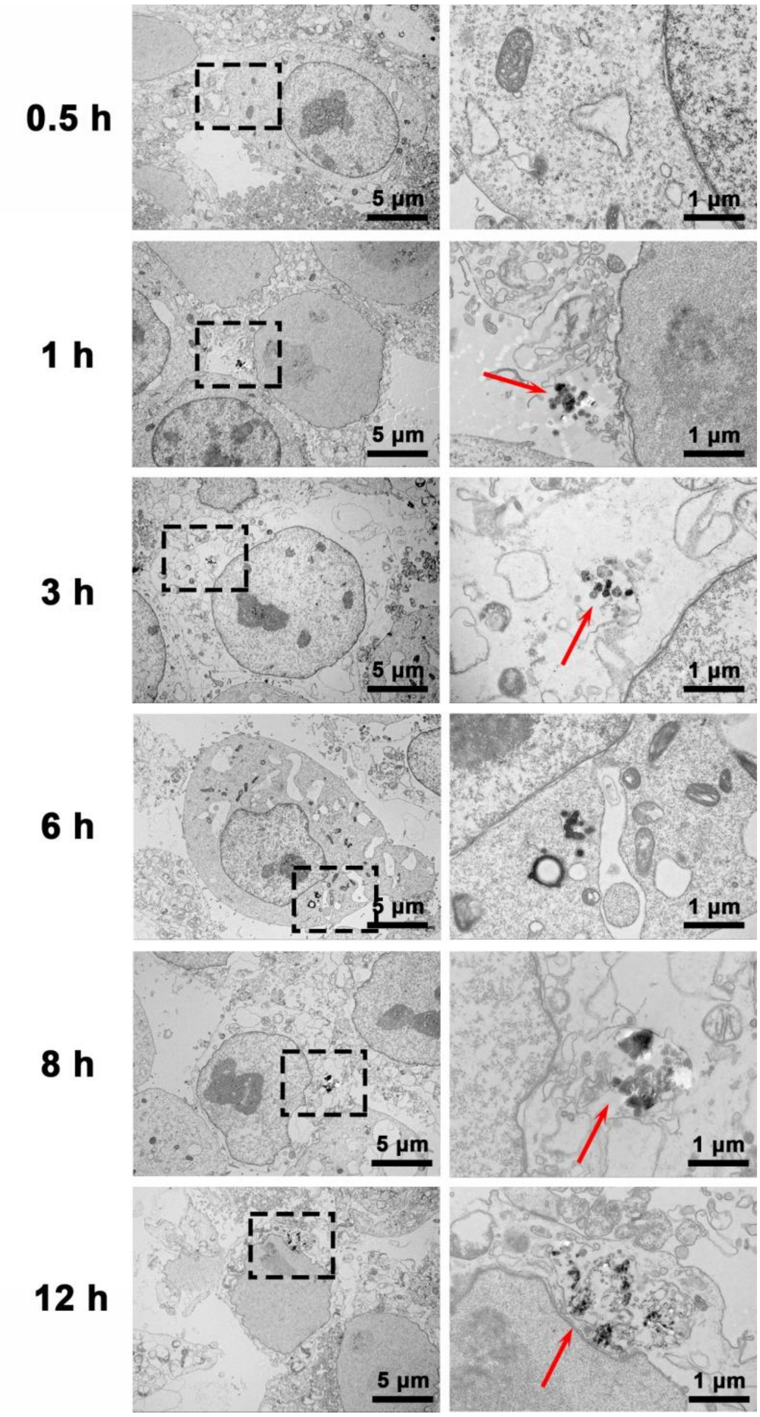

Figure S24. Bio–TEM images of 4T1 cells treated with MnDIG@PEG after incubating for 0.5, 1, 3, 6, 8, and 12 h at 37 °C, respectively.

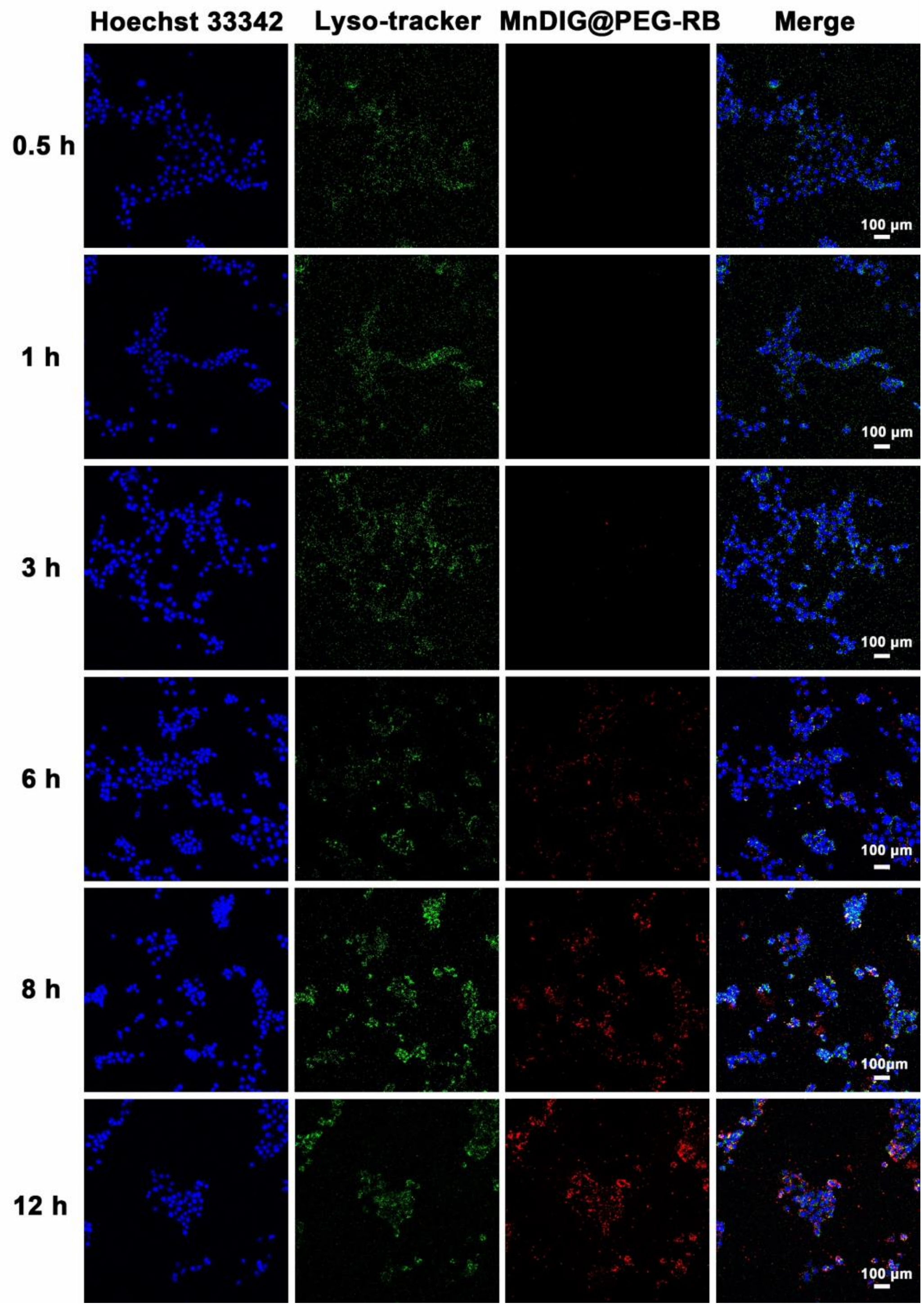

Figure S25. Confocal images of MnDIG@PEG-RB in 4T1 cells after incubating for 0.5, 1, 3, 6, 8, and 12 h at 37 °C, respectively. Scale bars: 100 μm.

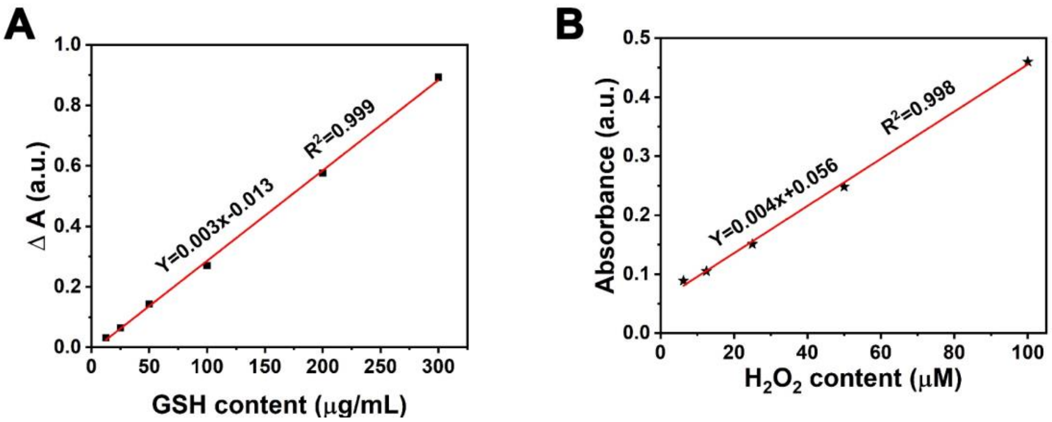

Figure S26. The standard curve of (A) GSH, (B) H_2O_2 *in vitro*.

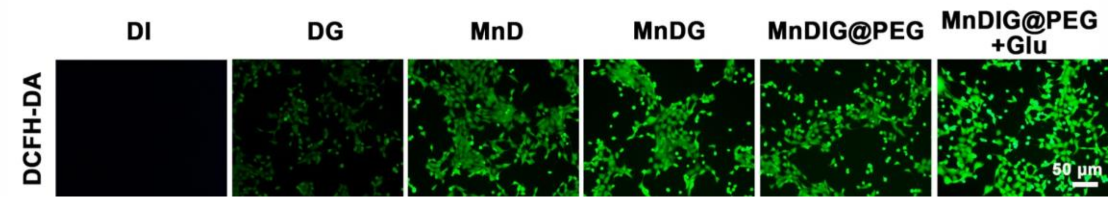

Figure S27. The detection of ROS in 4T1 cells by staining with DCFH-DA after different treatments.

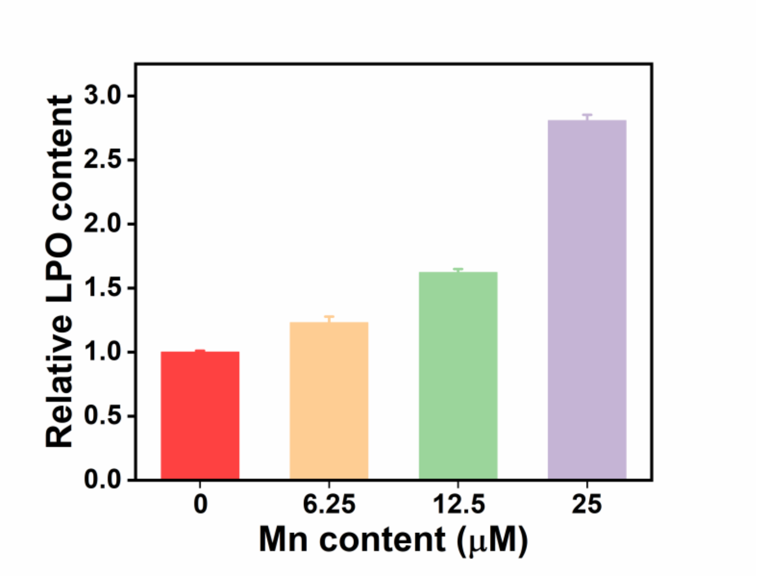

Figure S28. Intracellular LPO detections of 4T1 cells after incubation with different concentration of MnDIG@PEG.

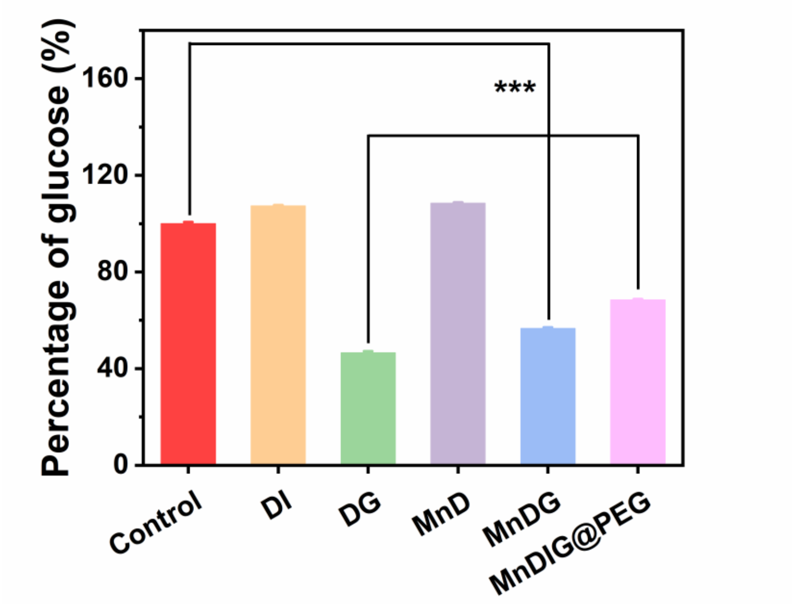

Figure S29. Intracellular glucose detections of 4T1 cells after 6 h of incubation with different samples (n = 3, mean ± SD).

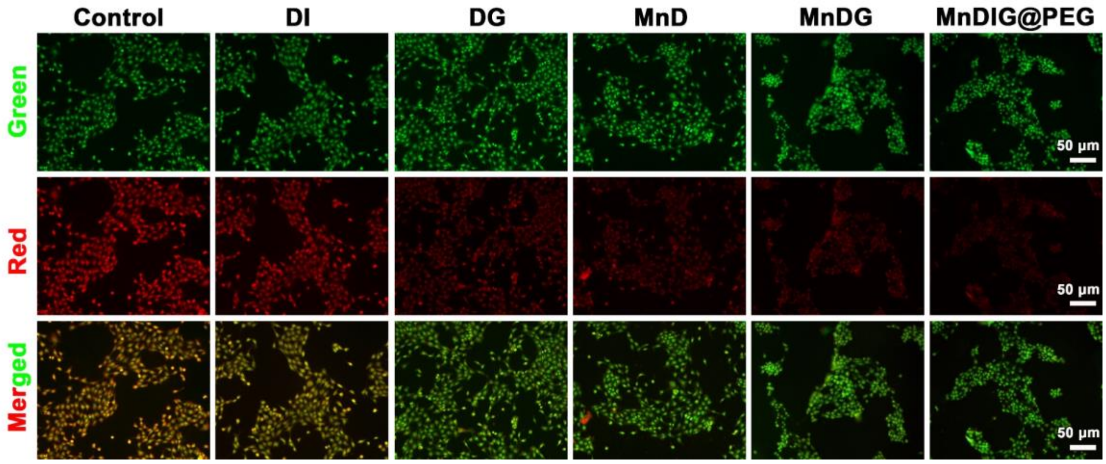

Figure S30. Fluorescent images of AO staining of 4T1 cells under different conditions.

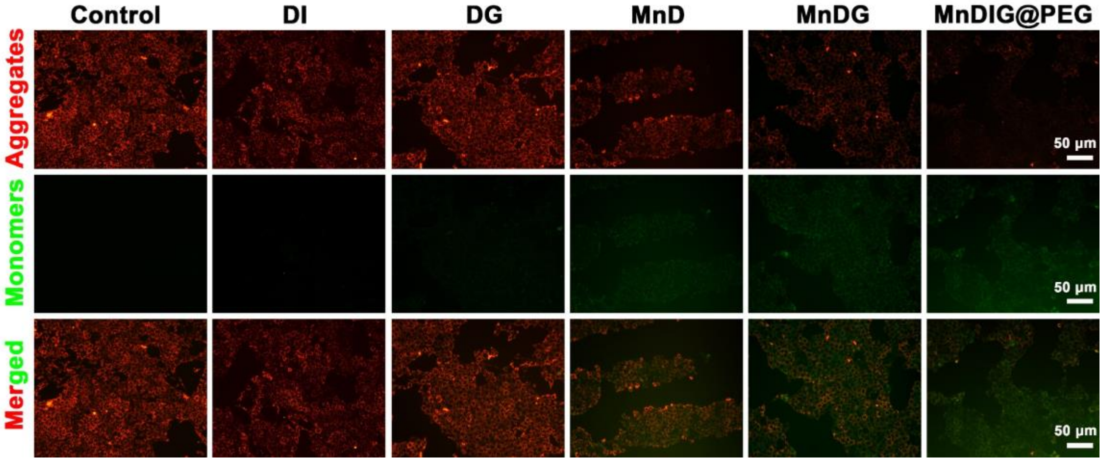

Figure S31. Fluorescent images of JC-1 assay of 4T1 cells under different conditions.

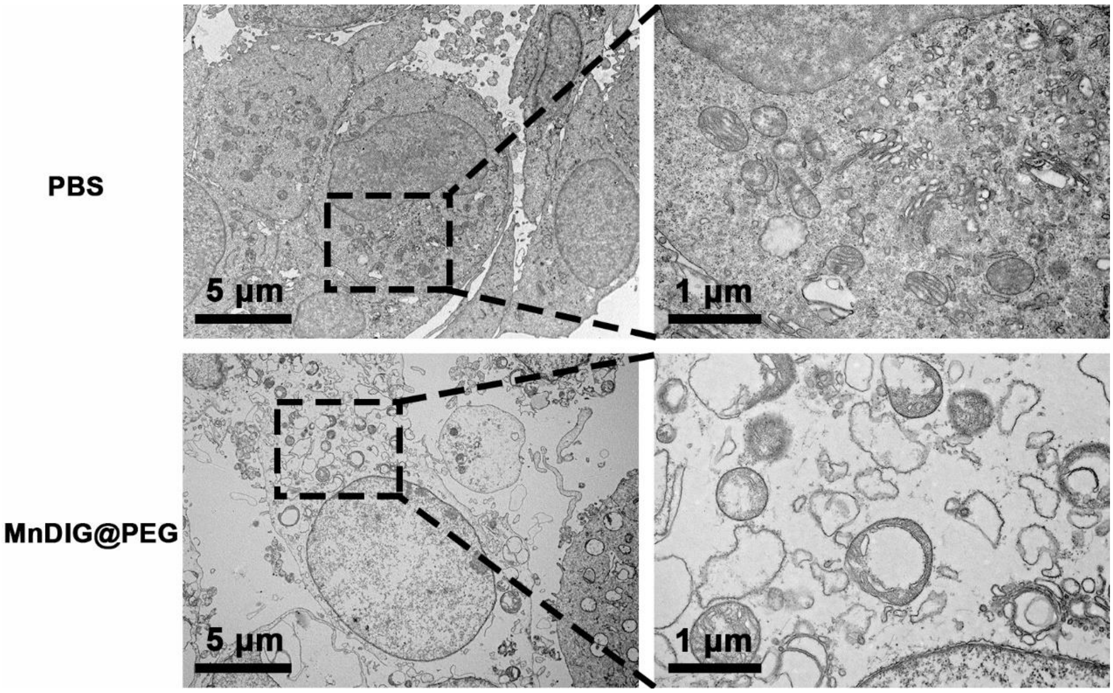

Figure S32. Bio–TEM images of 4T1 cells treated with PBS or MnDIG@PEG. An obvious shrunken mitochondria and decreased mitochondrial cristae could be observed, suggesting mitochondria damage happened.

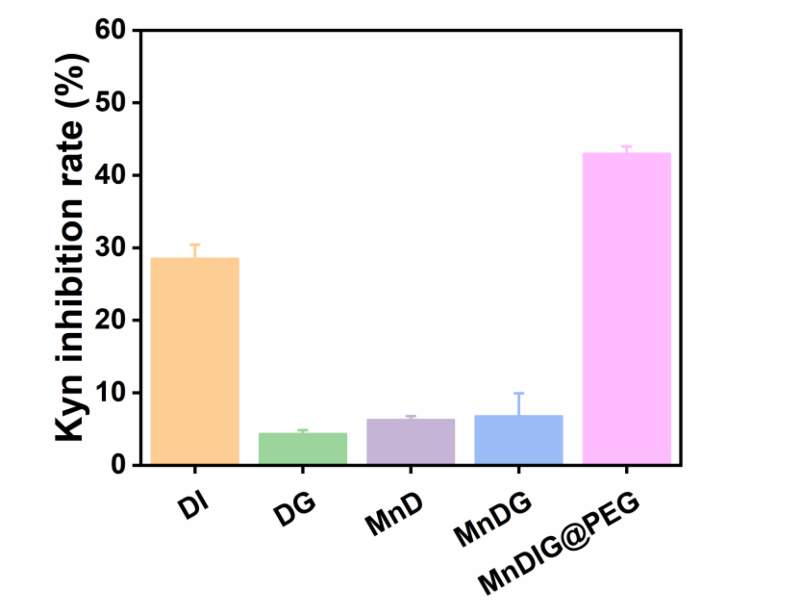

Figure S33. Intracellular Kyn inhibition rate of 4T1 cells after 12 h of incubation with different samples (n = 3, mean ± SD).

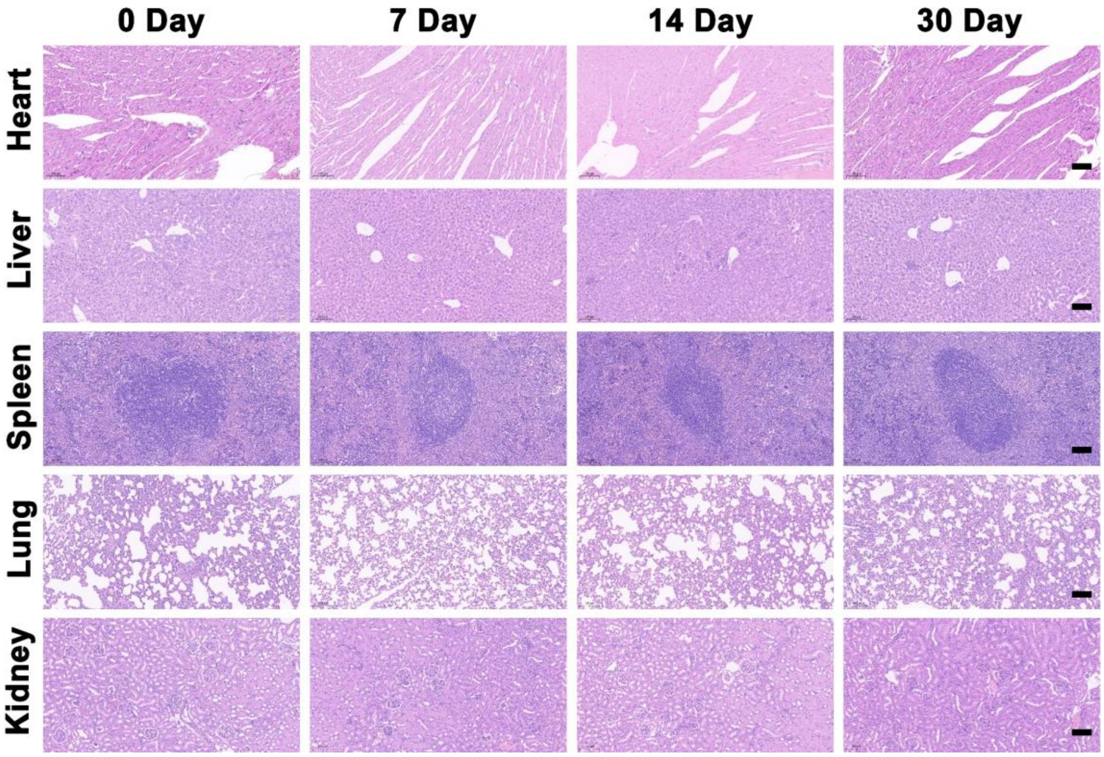

Figure S34. H&E staining images of major organs of tumor-bearing mice after treatment with MnDIG@PEG for 0, 7, 14, and 30 days, respectively. Scale bars: 100 μm.

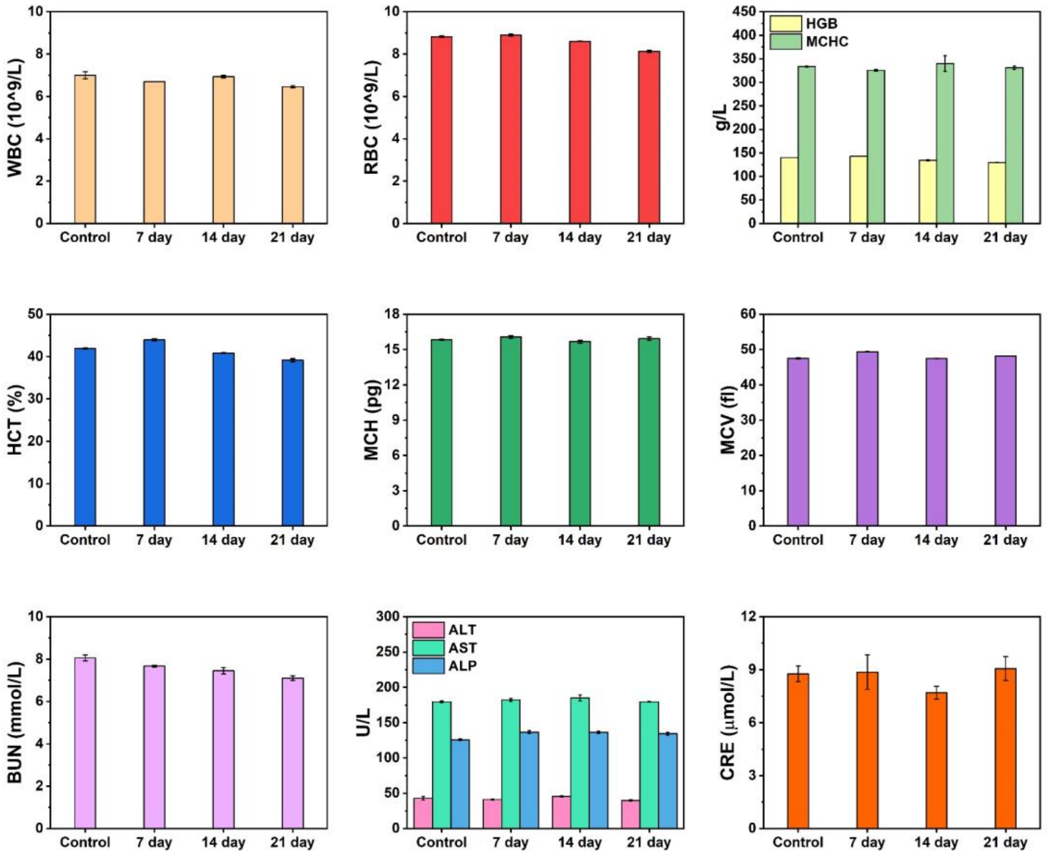

Figure S35. Blood biochemical and hematological analysis of the mice post-injection of MnDIG@PEG at given time-points (n = 3, mean ± SD).

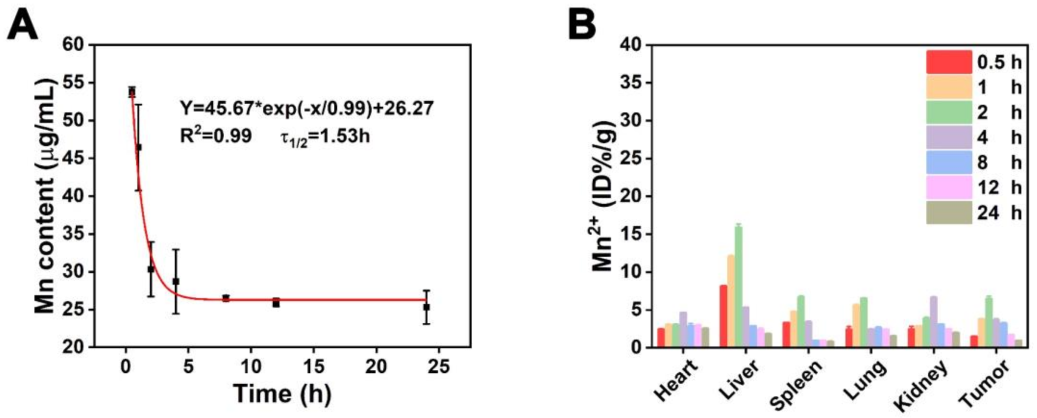

Figure S36. (A) The blood circulation curve of Mn^{2+} from intravenously injected MnDIG@PEG (n = 3, mean ± SD). (B) The bio-distribution of Mn^{2+} (injected dose (ID) % of Mn^{2+} per gram of tissues) in main tissues and tumor in 0.5, 1, 2, 4, 8, 12, and 24 h of intravenous administrations of MnDIG@PEG (n = 3, mean ± SD).

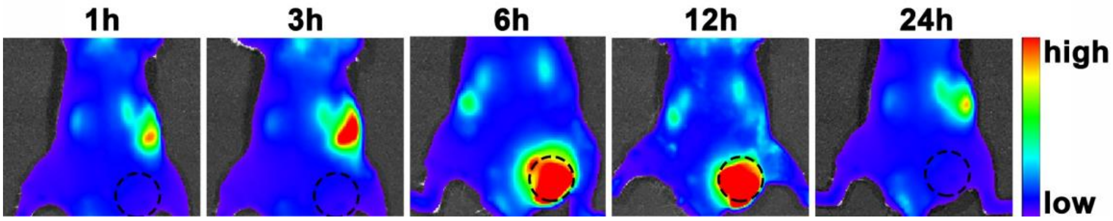

Figure S37. *In vivo* fluorescence imaging upon intravenously treated with Cy5-loaded MnDIG@PEG.

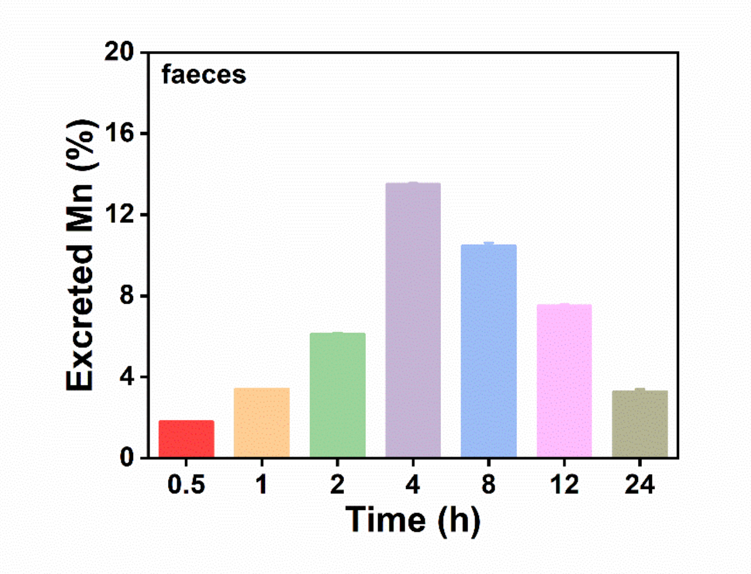

Figure S38. Mn excretion out of the mice body after the administration of MnDIG@PEG nanocomposites for different durations (n = 3, mean ± SD).

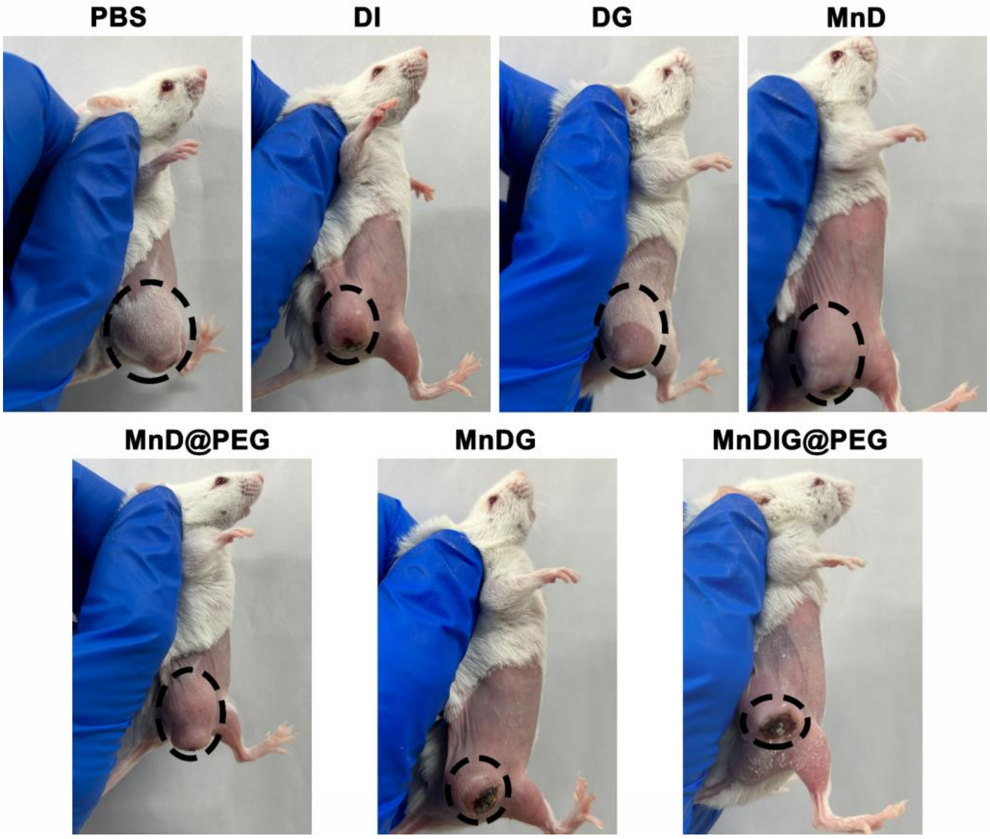

Figure S39. Representative images of tumor-bearing mice after 14 days treated with different samples.

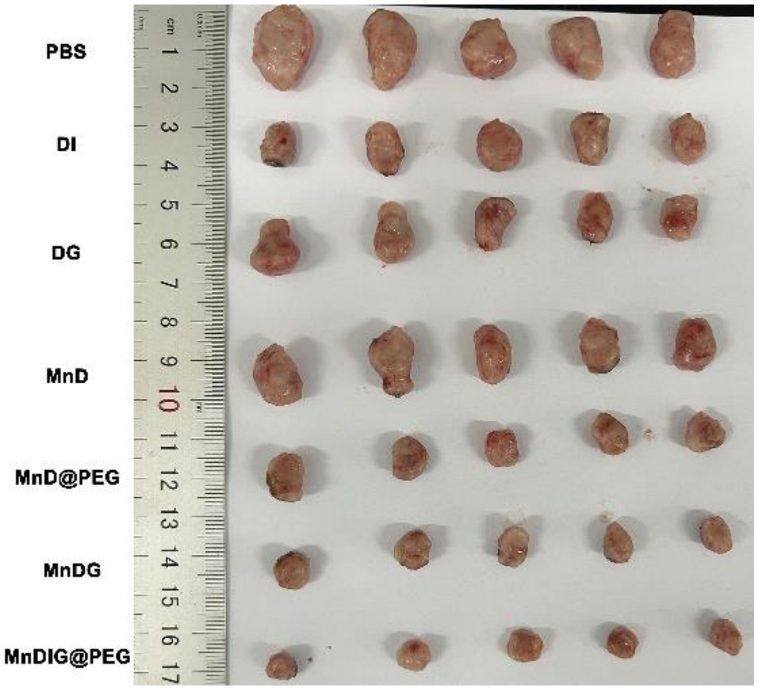

Figure S40. Representative images of the excised tumors after 14 days with different treatments.

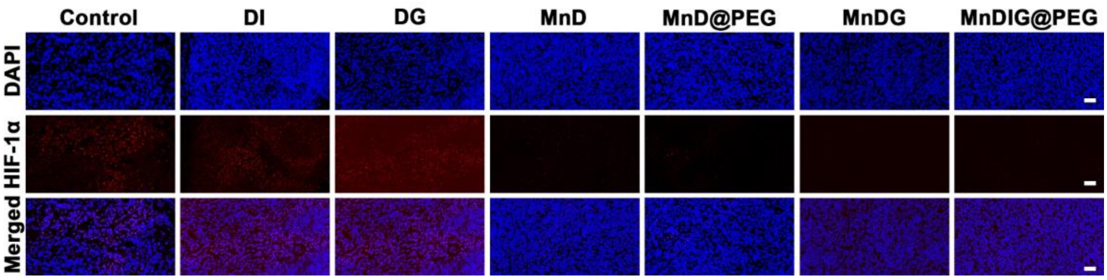

Figure S41. The HIF-1α staining images of tumors after treatment with different samples for 14 days, respectively. Scale bars: 50 µm.

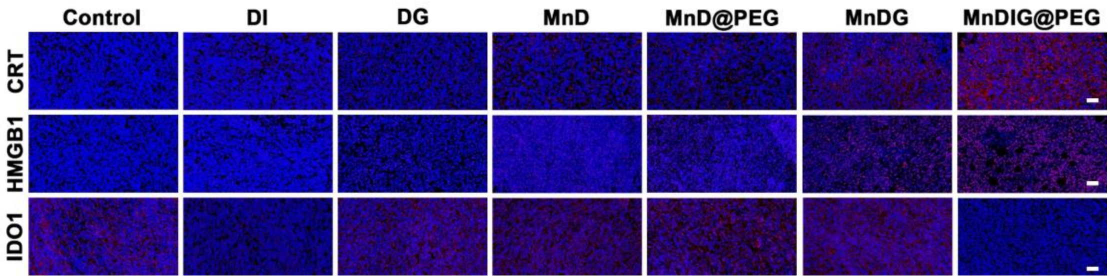

Figure S42. The CRT, HMGB1, and IDO1 staining images of tumors after treatment with different samples for 14 days, respectively. Scale bars: 50 μm.

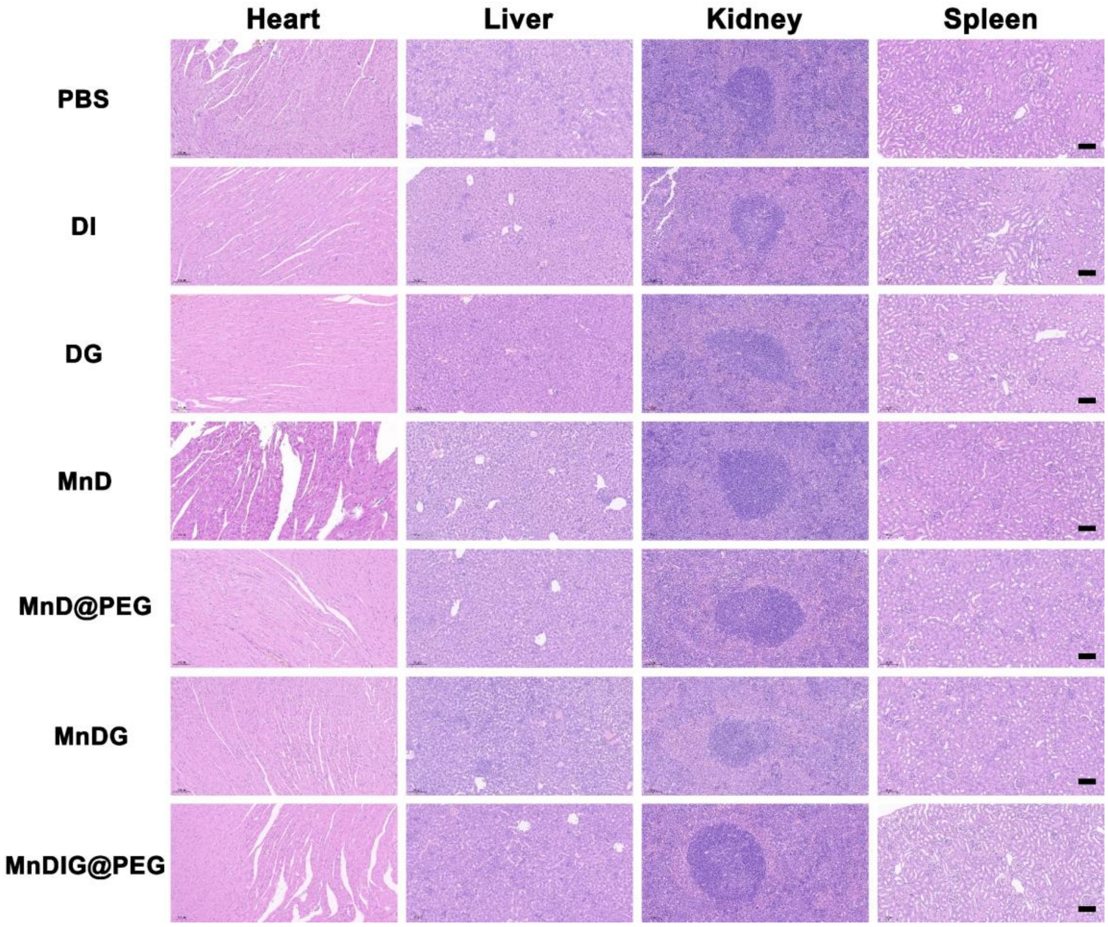

Figure S43. H&E staining images of major organs of tumor-bearing mice after treatment with different samples for 14 days, respectively. Scale bars: 100 μm.

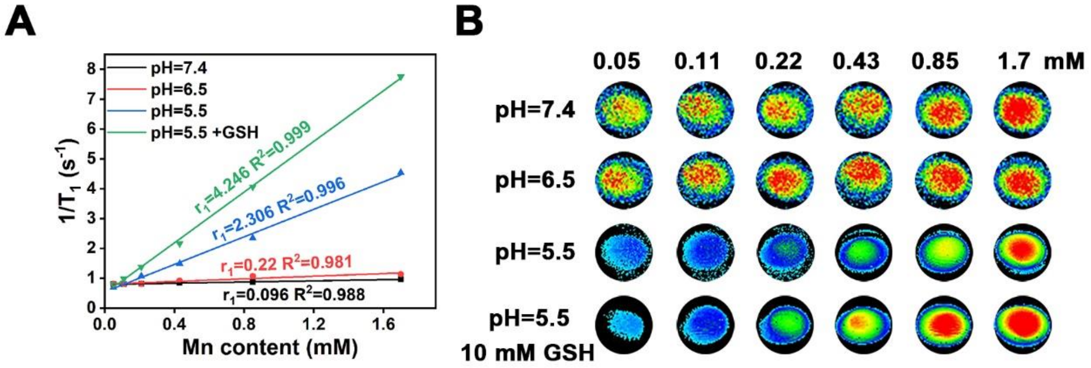

Figure S44. (A) Plots of *1/T*$_1$ *versus* concentration of MnDIG@PEG treated with different SBFs. (B) *In vitro* *T*$_1$-weighted MRI with different concentrations of MnDIG@PEG treated with different SBFs.

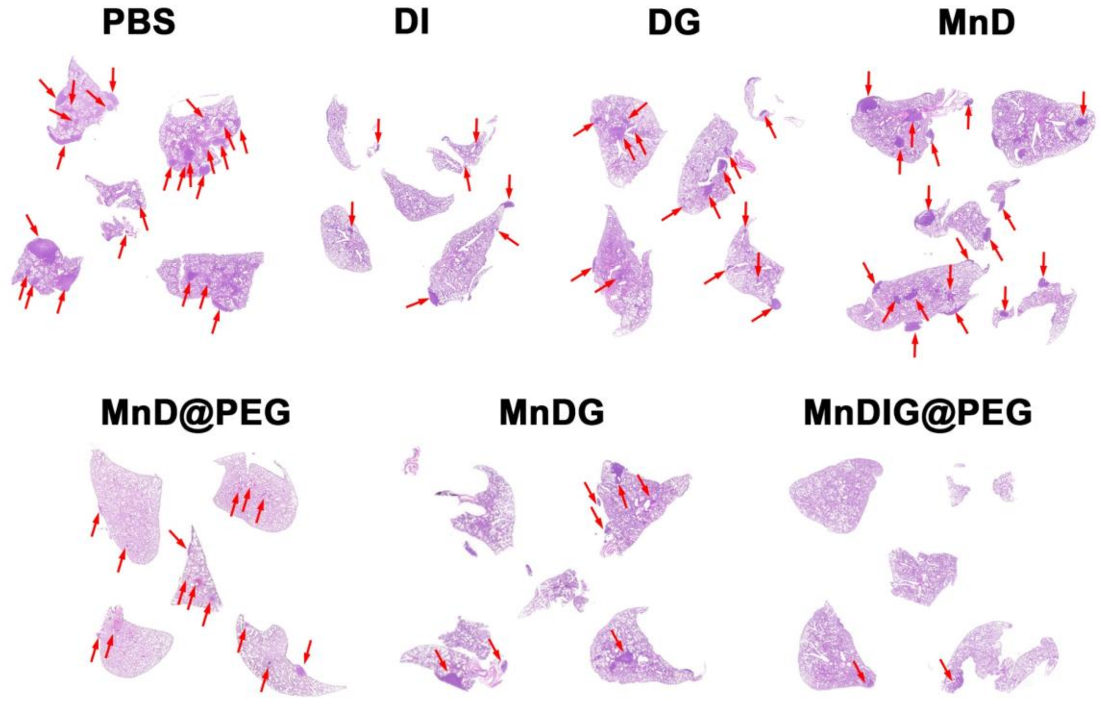

Figure S45. H&E staining of lungs in anti-metastatic studies.

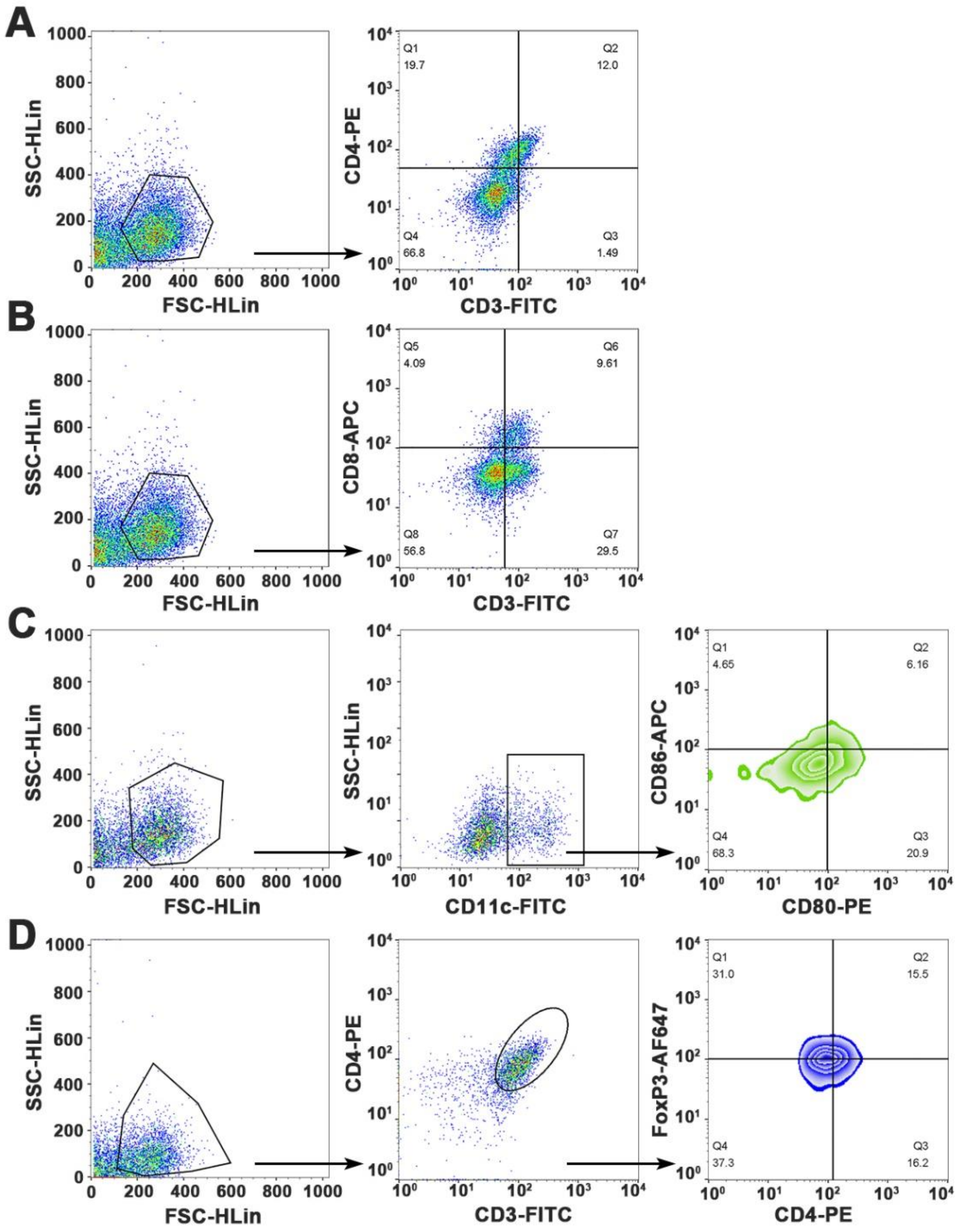

Figure S46. Gating strategy of (A) CD4$^+$ T cells, (B) CD8$^+$ T cells, (C) DCs, and (D) Treg cells collected from spleens for flow cytometry analysis.

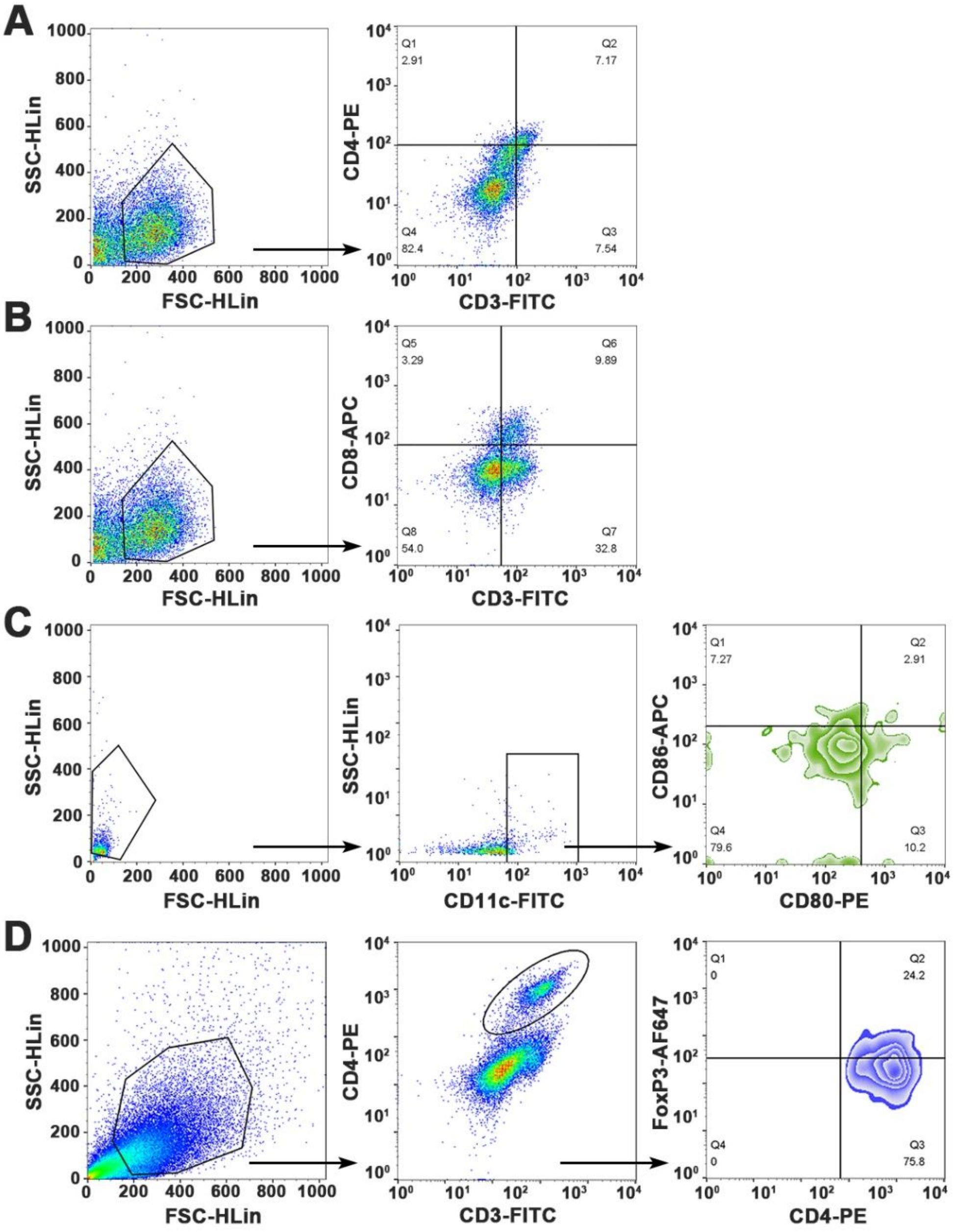

Figure S47. Gating strategy of (A) CD4+ T cells, (B) CD8+ T cells, (C) DCs, and (D) Treg cells collected from tumors for flow cytometry analysis.

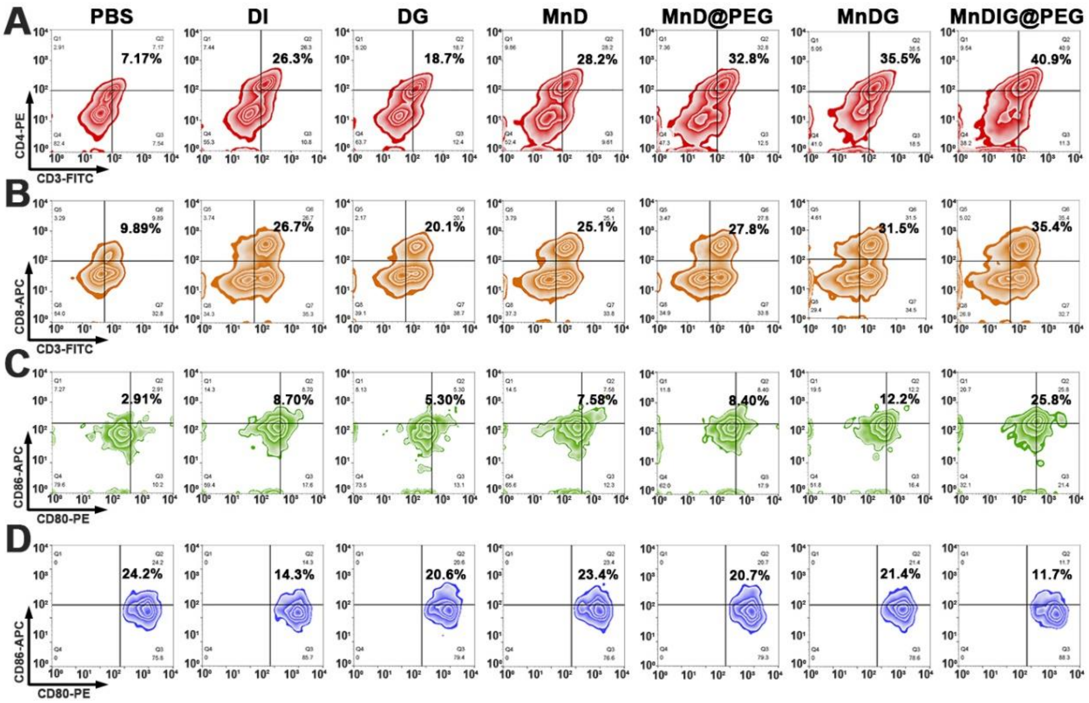

Figure S48. Flow cytometric analyses of the populations of (A) $CD4^+$ T cells, (B) $CD8^+$ T cells, (C) DC cells, and (D) Treg cells collected from tumors of tumor-bearing mice with different treatments.

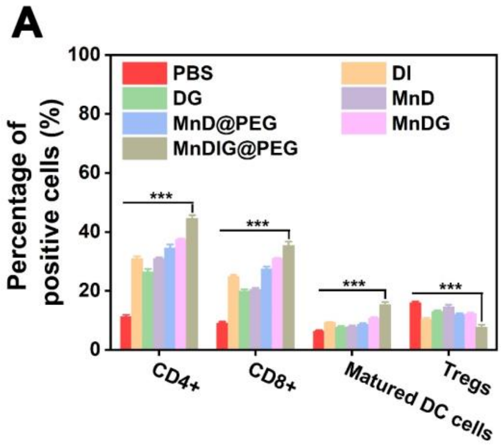

Figure S49. Quantitative analysis on the populations of $CD4^+$ T cells, $CD8^+$ T cells, DC cells, and Treg cells collected from (A) spleens, and (B) tumors of the 4T1 tumor-bearing mice with different treatments, respectively.
